# Supplementary material for: The transcription factor ChREBP Orchestrates liver carcinogenesis by coordinating the PI3K/AKT signaling and cancer metabolism
Source: Nat Commun. 2024 Feb 29;15:1879. doi: 10.1038/s41467-024-45548-w (PMC10904844; doi:10.1038/s41467-024-45548-w)
Supplement: Supplementary file 1 — Supplementary information [file 41467_2024_45548_MOESM1_ESM.pdf]

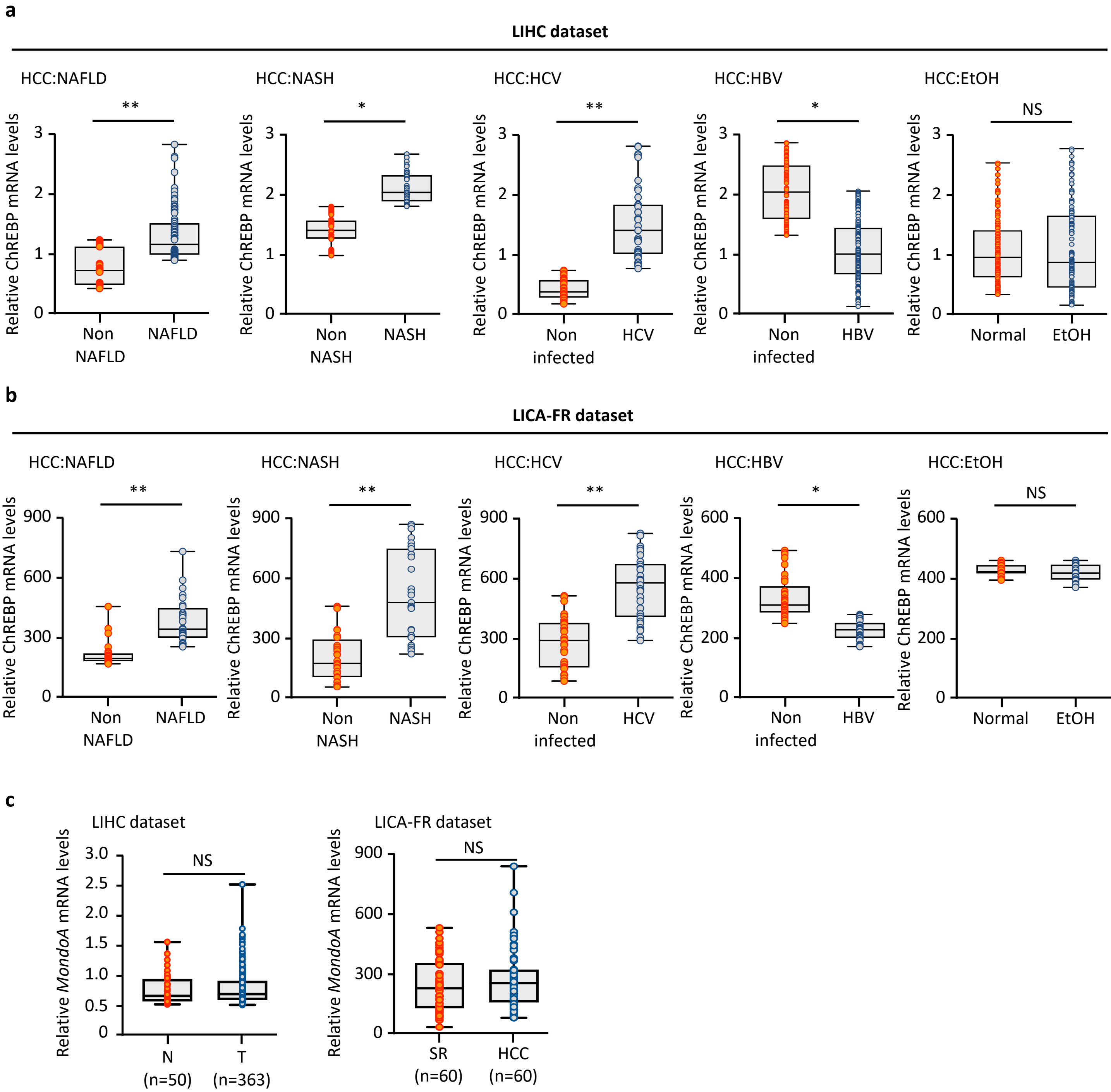
a
LIHC dataset
HCC:NAFLD
HCC:NASH
HCC:HCV
HCC:HBV
HCC:EtOH
Relative ChREBP mRNA levels
**
*
**
*
NS
0
1
2
3
Non NAFLD
NAFLD
Non NASH
NASH
Non infected
HCV
Non infected
HBV
Normal
EtOH
b
LICA-FR dataset
HCC:NAFLD
HCC:NASH
HCC:HCV
HCC:HBV
HCC:EtOH
Relative ChREBP mRNA levels
**
**
**
*
NS
0
300
600
900
0
200
400
600
Non NAFLD
NAFLD
Non NASH
NASH
Non infected
HCV
Non infected
HBV
Normal
EtOH
c
LIHC dataset
LICA-FR dataset
Relative MondoA mRNA levels
NS
NS
0
0.5
1.0
1.5
2.0
2.5
3.0
0
300
600
900
N (n=50)
T (n=363)
SR (n=60)
HCC (n=60)

Figure. S1

Supplementary Figure 1. ChREBP expression is increased in *human* HCC and signs tumors with poor prognosis. **(a and b)** Expression levels of ChREBP respectively in the LIHC **(a)** and LICA-FR **(b)** datasets depicting a positive correlation between its expression levels with chronic liver diseases including NAFLD, NASH and HCV infection. **(c)** Data mining of MondoA gene expression level between HCC (T) and normal liver tissues (N) from the LIHC and LICA-FR Oncomine datasets. For all box plots, the boundary of the box closest to zero indicates the 25 th percentile, a black line within the box marks the median, and the boundary of the box farthest from zero indicates the 75 th percentile. Whiskers above and below the box indicate the 10 th and 90 th percentiles. Points above and below the whiskers indicate outliers outside the 10 th and 90 th percentiles (statistical analysis can be found in the Source Data file). (a, b and c) Statistical analyses were made using unpaired two-sided Student's t-test. *$P < 0.05$. **$P < 0.01$. NS = non-significant.

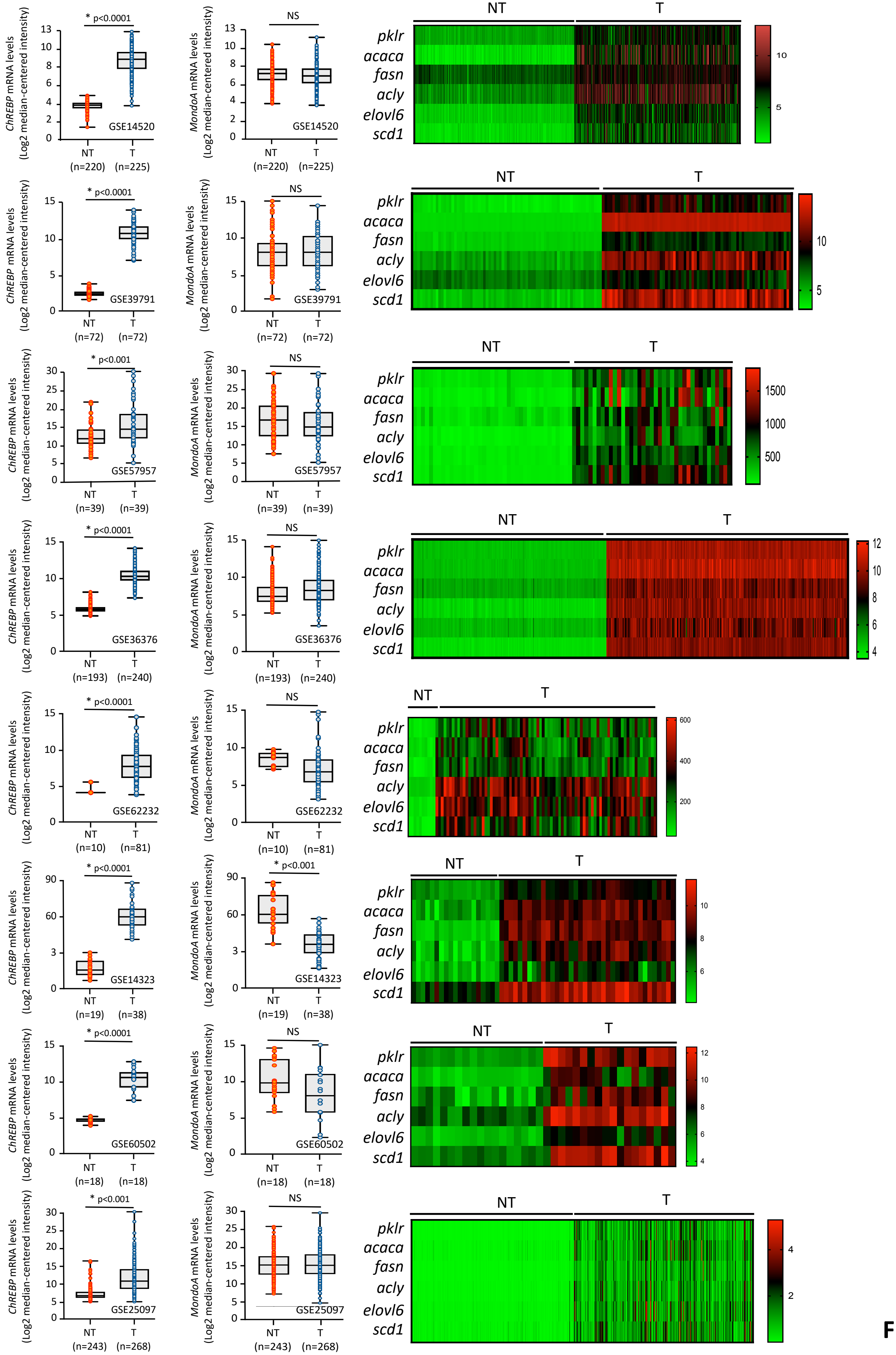
ChREBP mRNA levels (Log2 median-centered intensity)
MondoA mRNA levels (Log2 median-centered intensity)
* p<0.0001
* p<0.001
NS
GSE14520
NT (n=220)
T (n=225)
GSE39791
NT (n=72)
T (n=72)
GSE57957
NT (n=39)
T (n=39)
GSE36376
NT (n=193)
T (n=240)
GSE62232
NT (n=10)
T (n=81)
GSE14323
NT (n=19)
T (n=38)
GSE60502
NT (n=18)
T (n=18)
GSE25097
NT (n=243)
T (n=268)
NT
T
pklr
acaca
fasn
acly
elovl6
scd1

Figure. S2

Supplementary Figure 2. ChREBP expression is systematically increased in publicly available HCC *human* datasets. Expression levels of ChREBP, MondoA and ChREBP-regulated genes between normal/adjacent liver tissues and HCC tumors from 8 publicly available datasets (Table S1). For all box plots, the boundary of the box closest to zero indicates the 25 th percentile, a black line within the box marks the median, and the boundary of the box farthest from zero indicates the 75 th percentile. Whiskers above and below the box indicate the 10 th and 90 th percentiles. Points above and below the whiskers indicate outliers outside the 10 th and 90 th percentiles (statistical analysis can be found in the Source Data file). Statistical analyses were made using unpaired two-sided Student's t-test. *$P < 0.01$.

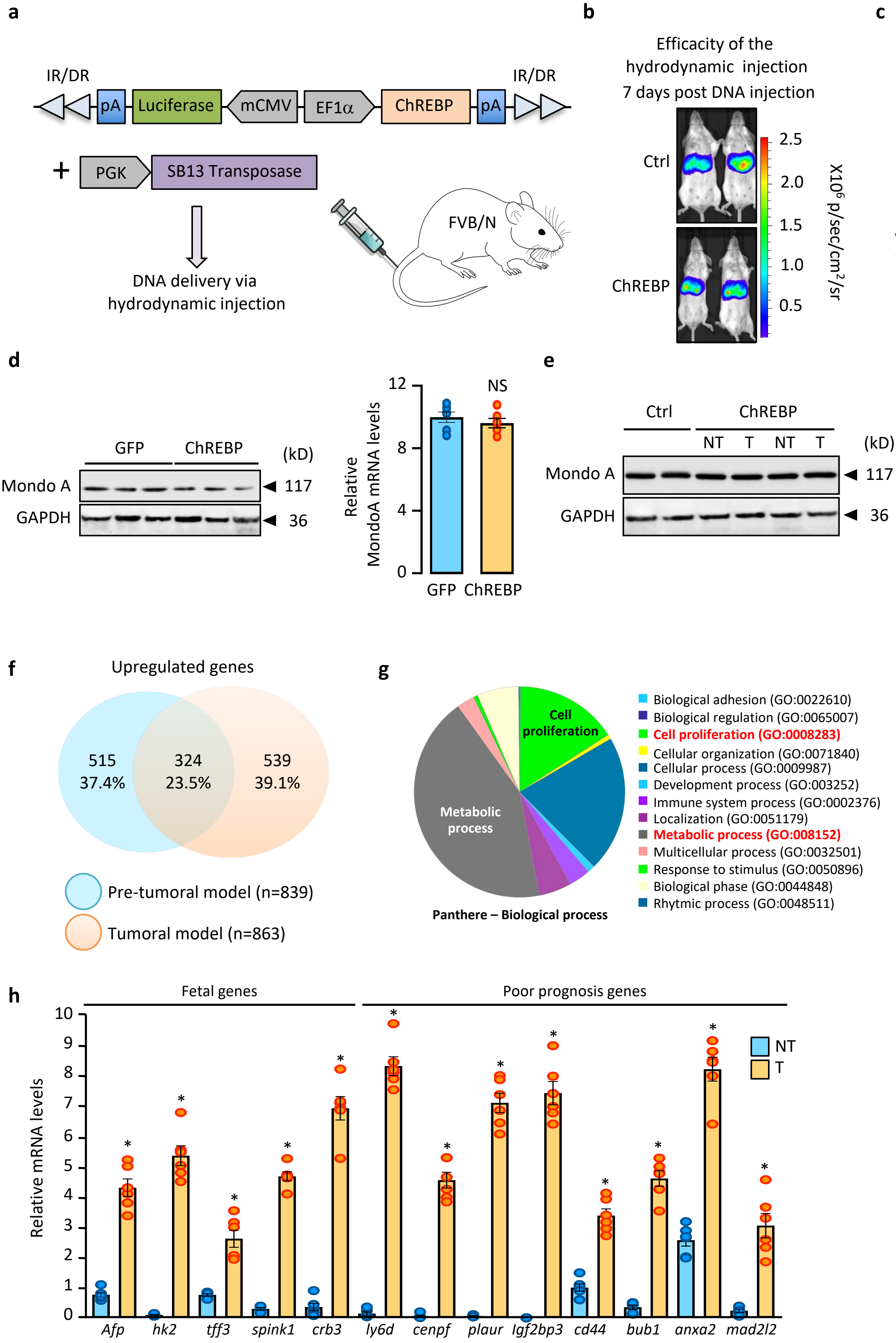

a
IR/DR
pA
Luciferase
mCMV
EF1α
ChREBP
pA
IR/DR
+
PGK
SB13 Transposase
DNA delivery via hydrodynamic injection
FVB/N
b
Efficacity of the hydrodynamic injection 7 days post DNA injection
Ctrl
ChREBP
X10^6 p/sec/cm^2/sr
c
% of Ki67 positive hepatocytes
NT
T
d
GFP
ChREBP
(kD)
Mondo A
GAPDH
117
36
Relative MondoA mRNA levels
NS
e
Ctrl
ChREBP
NT
T
Mondo A
GAPDH
f
Upregulated genes
515 37.4%
324 23.5%
539 39.1%
Pre-tumoral model (n=839)
Tumoral model (n=863)
g
Cell proliferation
Metabolic process
Panthere – Biological process
Biological adhesion (GO:0022610)
Biological regulation (GO:0065007)
Cell proliferation (GO:0008283)
Cellular organization (GO:0071840)
Cellular process (GO:0009987)
Development process (GO:003252)
Immune system process (GO:0002376)
Localization (GO:0051179)
Metabolic process (GO:008152)
Multicellular process (GO:0032501)
Response to stimulus (GO:0050896)
Biological phase (GO:0044848)
Rhytmic process (GO:0048511)
h
Fetal genes
Poor prognosis genes
Relative mRNA levels
Afp
hk2
tff3
spink1
crb3
ly6d
cenpf
plaur
Igf2bp3
cd44
bub1
anxa2
mad2l2

Figure. S3

Supplementary Figure 3. Stable ChREBP overexpression promotes HCC initiation and development in *mice*. **(a)** Schematic representation of the vectors used for the hydrodynamic injections: PGK/SB13 transposase, pT3/Caggs-luciferase (empty vector / Ctrl) and pT3/Caggs-ChREBP-luciferase (in-one). **(b)** Efficacity of the hydrodynamic injection measured by bioluminescence imaging 7 days post treatment (n = 10 biologically independent *mice* per group). **(c)** Quantification of Ki67 immunostaining shown in Figure 2g (n = 10 biologically independent *mice* per group). **(d)** Expression profile of MondoA protein content and relative MondoA gene expression corrected to TBP in ChREBP overexpressing *mice* (n = 6 biologically independent *mice* per group). **(e)** Expression profile of MondoA protein content and relative MondoA gene expression corrected to TBP in ChREBP tumors (n = 6 biologically independent *mice* per group). **(f)** Venn diagram of overlap between genes that are differentially upregulated in the liver of *mice* overexpressing ChREBP for 3 weeks (pre-malignant) and in ChREBP tumors (n = 12 biologically independent *mice* per group). **(g)** Genes that were commonly upregulated in the liver of *mice* overexpressing ChREBP for 3 weeks and in ChREBP tumors were subjected to gene ontology enrichment analysis. Pie charts represent stepwise hierarchical tree drill down of significantly enriched terms under the heading biological process. **(h)** Relative expression of genes expressed in poor prognosis HCC measured in ChREBP tumors. The expression of specific gene was normalized to TBP (TATA binding protein) mRNA levels (n = 6 biologically independent *mice* per group). All error bars represent mean ± SEM. (c, d, e, h) Statistical analyses were made using unpaired two-sided Student's t-test $^{*}P < 0.001$ NT vs T, NS = Non-Significant.

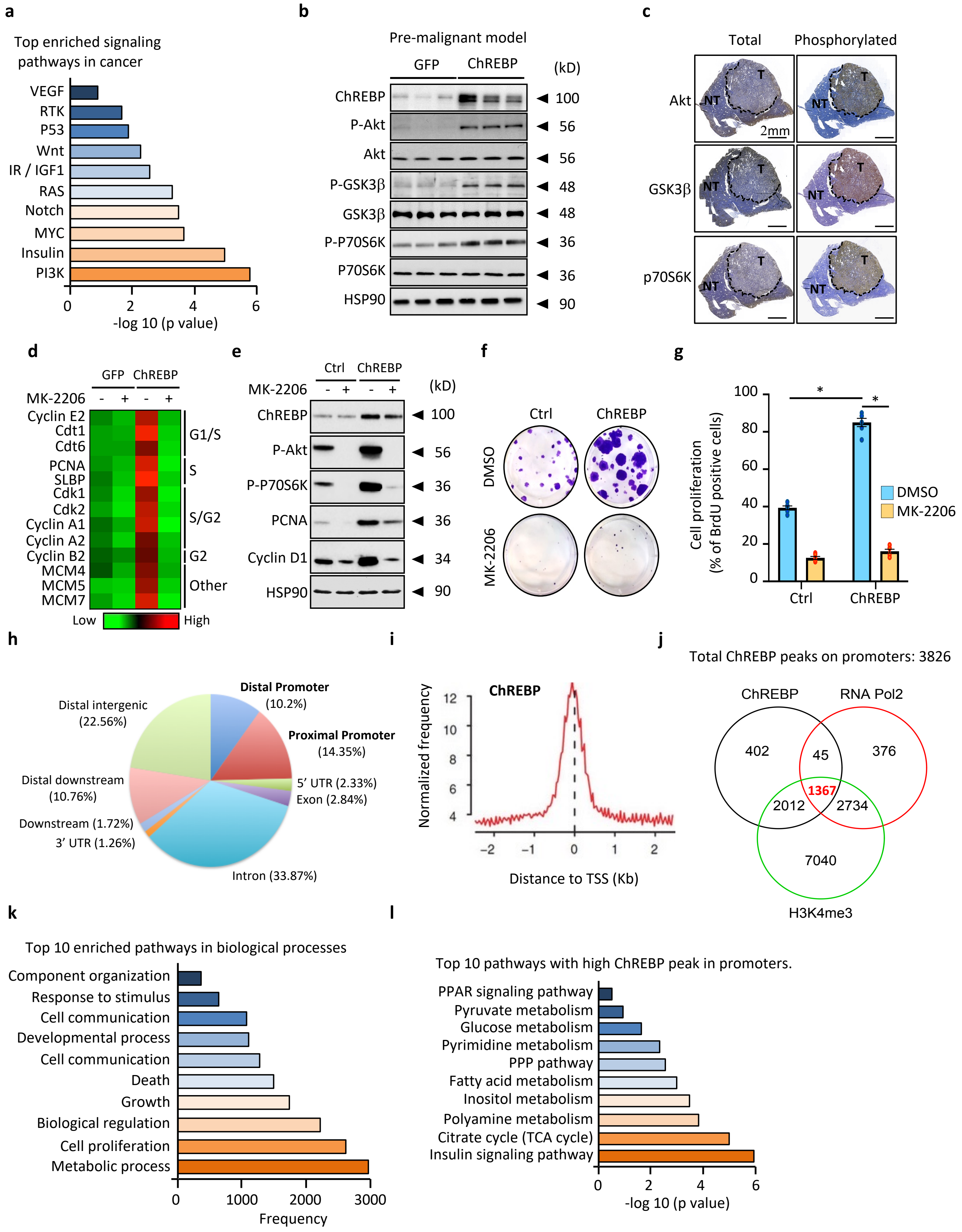
a
Top enriched signaling pathways in cancer
VEGF
RTK
P53
Wnt
IR / IGF1
RAS
Notch
MYC
Insulin
PI3K
0 2 4 6
-log 10 (p value)
b
Pre-malignant model
GFP ChREBP
(kD)
ChREBP 100
P-Akt 56
Akt 56
P-GSK3β 48
GSK3β 48
P-P70S6K 36
P70S6K 36
HSP90 90
c
Total Phosphorylated
Akt
GSK3β
p70S6K
T
NT
2mm
d
GFP ChREBP
MK-2206 - + - +
Cyclin E2
Cdt1
Cdt6
PCNA
SLBP
Cdk1
Cdk2
Cyclin A1
Cyclin A2
Cyclin B2
MCM4
MCM5
MCM7
G1/S
S
S/G2
G2
Other
Low High
e
Ctrl ChREBP
MK-2206 - + - +
(kD)
ChREBP 100
P-Akt 56
P-P70S6K 36
PCNA 36
Cyclin D1 34
HSP90 90
f
Ctrl ChREBP
DMSO
MK-2206
g
Cell proliferation (% of BrdU positive cells)
0 20 40 60 80 100
*
*
DMSO
MK-2206
Ctrl ChREBP
h
Distal Promoter (10.2%)
Proximal Promoter (14.35%)
5′ UTR (2.33%)
Exon (2.84%)
Intron (33.87%)
3′ UTR (1.26%)
Downstream (1.72%)
Distal downstream (10.76%)
Distal intergenic (22.56%)
i
ChREBP
Normalized frequency
4 6 8 10 12
-2 -1 0 1 2
Distance to TSS (Kb)
j
Total ChREBP peaks on promoters: 3826
ChREBP
RNA Pol2
H3K4me3
402
45
376
1367
2012
2734
7040
k
Top 10 enriched pathways in biological processes
Component organization
Response to stimulus
Cell communication
Developmental process
Cell communication
Death
Growth
Biological regulation
Cell proliferation
Metabolic process
0 1000 2000 3000
Frequency
l
Top 10 pathways with high ChREBP peak in promoters.
PPAR signaling pathway
Pyruvate metabolism
Glucose metabolism
Pyrimidine metabolism
PPP pathway
Fatty acid metabolism
Inositol metabolism
Polyamine metabolism
Citrate cycle (TCA cycle)
Insulin signaling pathway
0 2 4 6
-log 10 (p value)

Figure. S4

Supplementary Figure 4. PI3K/AKT signaling mediates ChREBP pro-proliferative effect. **(a)** Top ten enriched signaling pathways in ChREBP tumors (n = 12 biologically independent *mice* per group). The list of differentially expressed genes under the term PI3K signaling can be found in supplementary table 6. **(b)** Representative Western blot analysis of the activity of the PI3K/AKT signaling in the pre-malignant model (n = 10 biologically independent *mice* per group). **(c)** Representative immuno-staining evaluating levels of AKT, GSK3β and p70S6K phosphorylation in ChREBP tumors (T) compared to non-tumoral tissue (NT) (n = 20 biologically independent *mice* per group). Scale bar = 2 mm. **(d)** Mice, injected with either GFP or ChREBP overexpressing adenovirus, were treated with or without MK-2206 (120 mg/kg) for 3 weeks. Heatmap presenting the expression of cell cycle genes (n = 12 biologically independent *mice* per group). **(e-g)** Huh7 cells, stably overexpressing ChREBP, were treated with DMSO or MK-2206 (100 nM) for 24h. **(e)** Representative Western blot analysis of the activity of the PI3K/AKT signaling and cell cycle (n =3 independent experiments). **(f)** Representative clonogenic assay shown (n =3 independent experiments). **(g)** Cell proliferation index determined by measuring the % of BrdU positive cells (n =3 independent experiments). **(h)** Genomic distribution of ChREBP peaks after ChIP-sequencing experiments from primary cultured hepatocytes incubated with 100 nM insulin and 25 mM glucose for 24 h. **(i)** Tag density plots displaying ChREBP tags distribution relative to the transcriptional start site (TSS). **(j)** Venn diagrams showing overlap between ChREBP-bound, RNA polII-bound and H3K4me3-marked promoters. **(k)** Top ten enriched biological processes from gene ontology analysis performed on identified ChREBP-target genes. **(l)** Top enriched pathways for the top 1000 genes with high ChREBP peak intensity within promoters. All error bars represent mean ± SEM. (g) Statistical analyses were made using two-way ANOVA and Tukey's multiple-comparisons test. * $P < 0.01$.

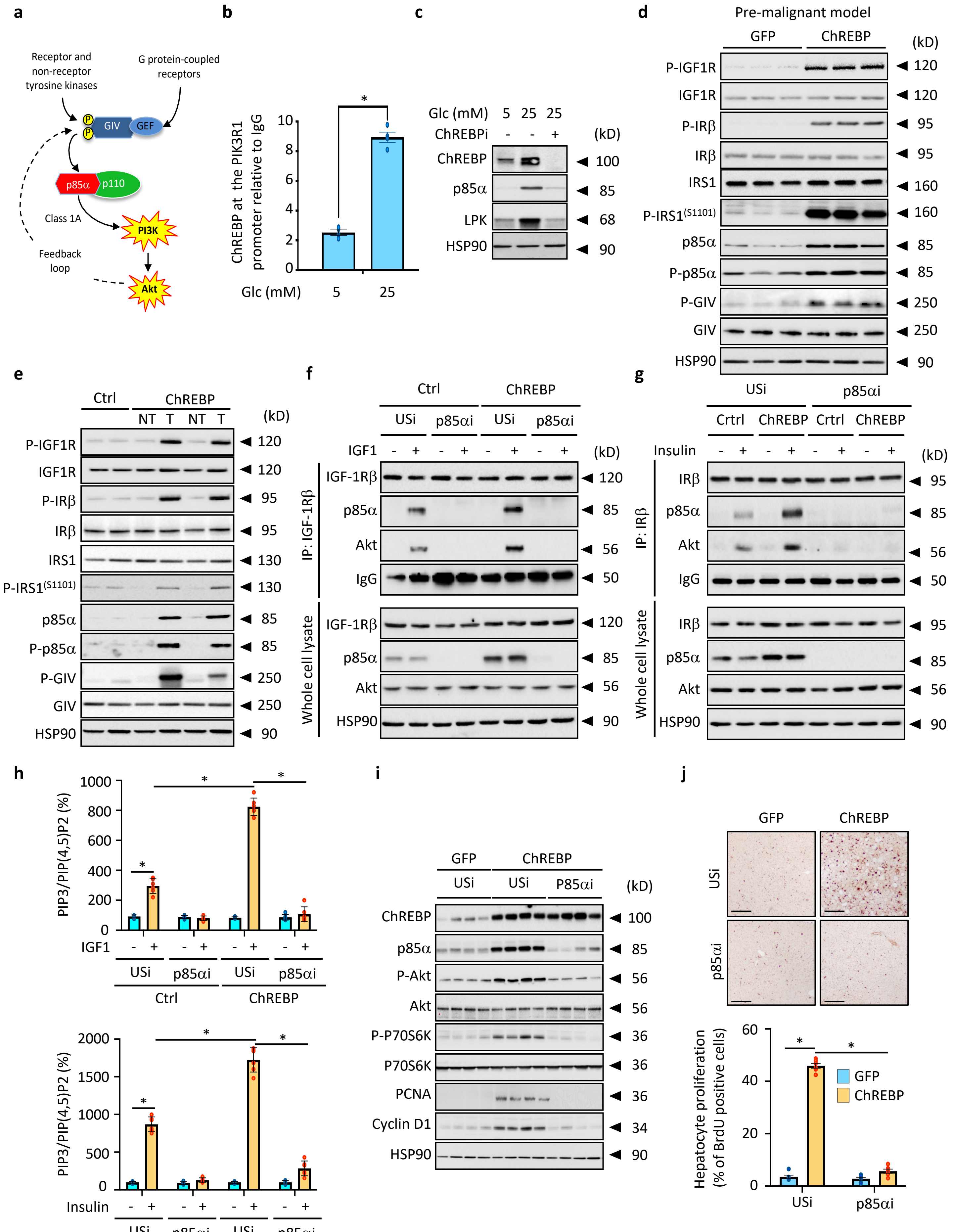
a
Receptor and non-receptor tyrosine kinases
G protein-coupled receptors
P
GIV
GEF
p85α
p110
Class 1A
PI3K
Feedback loop
Akt
b
ChREBP at the PIK3R1 promoter relative to IgG
Glc (mM)
5
25
c
Glc (mM) 5 25 25
ChREBPi - - +
(kD)
ChREBP 100
p85α 85
LPK 68
HSP90 90
d
Pre-malignant model
GFP
ChREBP
(kD)
P-IGF1R 120
IGF1R 120
P-IRβ 95
IRβ 95
IRS1 160
P-IRS1(S1101) 160
p85α 85
P-p85α 85
P-GIV 250
GIV 250
HSP90 90
e
Ctrl
ChREBP
NT T NT T
(kD)
P-IGF1R 120
IGF1R 120
P-IRβ 95
IRβ 95
IRS1 130
P-IRS1(S1101) 130
p85α 85
P-p85α 85
P-GIV 250
GIV 250
HSP90 90
f
Ctrl
ChREBP
USi p85αi USi p85αi
IGF1 - + - + - + - +
(kD)
IP: IGF-1Rβ
IGF-1Rβ 120
p85α 85
Akt 56
IgG 50
Whole cell lysate
IGF-1Rβ 120
p85α 85
Akt 56
HSP90 90
g
USi
p85αi
Crtrl ChREBP Crtrl ChREBP
Insulin - + - + - + - +
(kD)
IP: IRβ
IRβ 95
p85α 85
Akt 56
IgG 50
Whole cell lysate
IRβ 95
p85α 85
Akt 56
HSP90 90
h
PIP3/PIP(4,5)P2 (%)
IGF1
USi p85αi USi p85αi
Ctrl
ChREBP
Insulin
i
GFP
ChREBP
USi USi P85αi
(kD)
ChREBP 100
p85α 85
P-Akt 56
Akt 56
P-P70S6K 36
P70S6K 36
PCNA 36
Cyclin D1 34
HSP90 90
j
GFP
ChREBP
USi
p85αi
Hepatocyte proliferation (% of BrdU positive cells)
GFP
ChREBP
USi
p85αi

Figure. S5

Supplementary Figure 5. p85α drives the stimulatory effects of ChREBP on PI3K/AKT signaling and hepatocyte proliferation. **(a)** Schematic representation of the role of p85α in controlling PI3K/AKT signaling. **(b)** ChIP experiments measuring ChREBP occupancy at the *Pik3r1* promoter relative to IgG controls in response to glucose stimulation (5 or 25 mM for 24h) in cultured hepatocytes (n = 4 independent experiments). **(c)**. ChREBP expression was inhibited in cultured hepatocytes incubated with 5 or 25 mM glucose and with 100 nM insulin for 24h. Representative Western blot of ChREBP and p85α expression is shown (n = 4 independent experiments). **(d)** *Mice*, injected with either GFP or ChREBP overexpressing adenovirus, were studied 3 weeks later. Representative Western blot analysis depicting IGF1R, IR and p85α activity (n = 10 biologically independent *mice* per group). **(e)** Representative Western blot analysis depicting IGF1R, IR and p85α activity in ChREBP overexpressing tumors (n = 10 biologically independent *mice* per group). **(f and g)** p85α stabilizes activated IGF1R or IR receptors / AKT complexes at the plasma membrane to increase PI3K activity in response to ChREBP overexpression. P85α expression was stably inhibited, through Crispr/Cas9 approach in Huh7 cell line overexpressing ChREBP. IGF1R **(f)** or IR **(g)**, immunoprecipitated from IGF1 (10 nM, 5 minutes) or insulin (10 nM, 5 minutes) stimulated Huh7 cells, were immunoblotted for IGF1R, IR, p85α and AKT. (n = 3 independent experiments). **(h)** PI3K activity was determined by measuring the ratio between PIP3 and PI(4,5)P2 levels in Huh7 cells stably overexpressing ChREBP. Huh7 cells were stimulated with either IGF1 (10 nM) or insulin (10 nM) for 5 minutes (n = 3 independent experiments). **(i and j)** P85α expression was inhibited, through adenoviral-mediated shRNA delivery, in the liver of mice overexpressing ChREBP. **(i)** Representative Western blot analysis of PI3K/AKT signaling and cell cycle (n = 10 biologically independent *mice* per group). **(j)** Representative staining of liver sections with BrdU (top panel). Quantification of the BrdU staining is shown (bottom panel) (n = 10 biologically independent *mice* per group). Scale bars = 100 µm. All error bars represent mean ± SEM. (b) Statistical analyses were made using unpaired two-sided Student's t-test *P < 0.001. (h, j) Statistical analyses were made using two-way ANOVA and Tukey's multiple-comparisons test. * P < 0.01.

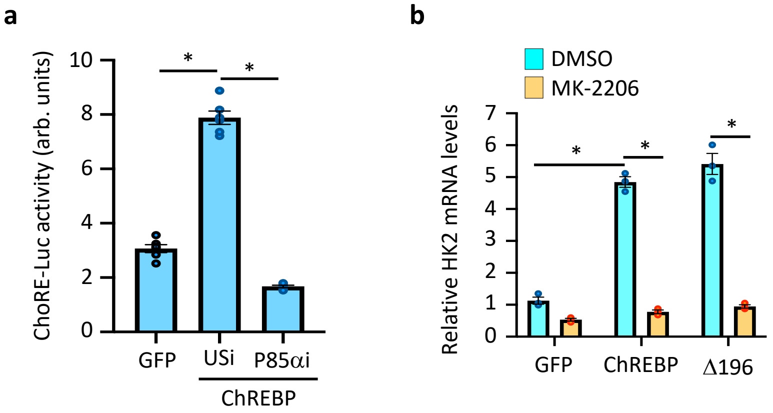

Supplementary Figure 6. Hexokinase 2 is a part of an amplification loop linking glucose mediated ChREBP activation with enhanced PI3K/AKT signaling. **(a)** C57BL6/J male *mice* were injected with either GFP or ChREBP overexpressing adenovirus. Simultaneously, P85α expression was also inhibited through adenoviral-mediated shRNA delivery. *Mice* were study 3 weeks later. **(a)** Quantification of ChoRE-Luc activity shown in Figure 5a (n = 6 biologically independent *mice* per group). **(b)** FLAG tagged WT or HA tagged Δ196 isoforms of ChREBP were overexpressed in Huh7 cells. Cells were then treated with 100 nM of MK-2206 for 24h. **(b)** HK2 expression relative to the TBP gene expression (n = 3 independent experiments). All error bars represent mean ± SEM. (a and b) Statistical analyses were made using two-way ANOVA and Tukey's multiple-comparisons test. * P < 0.01.

Figure. S6

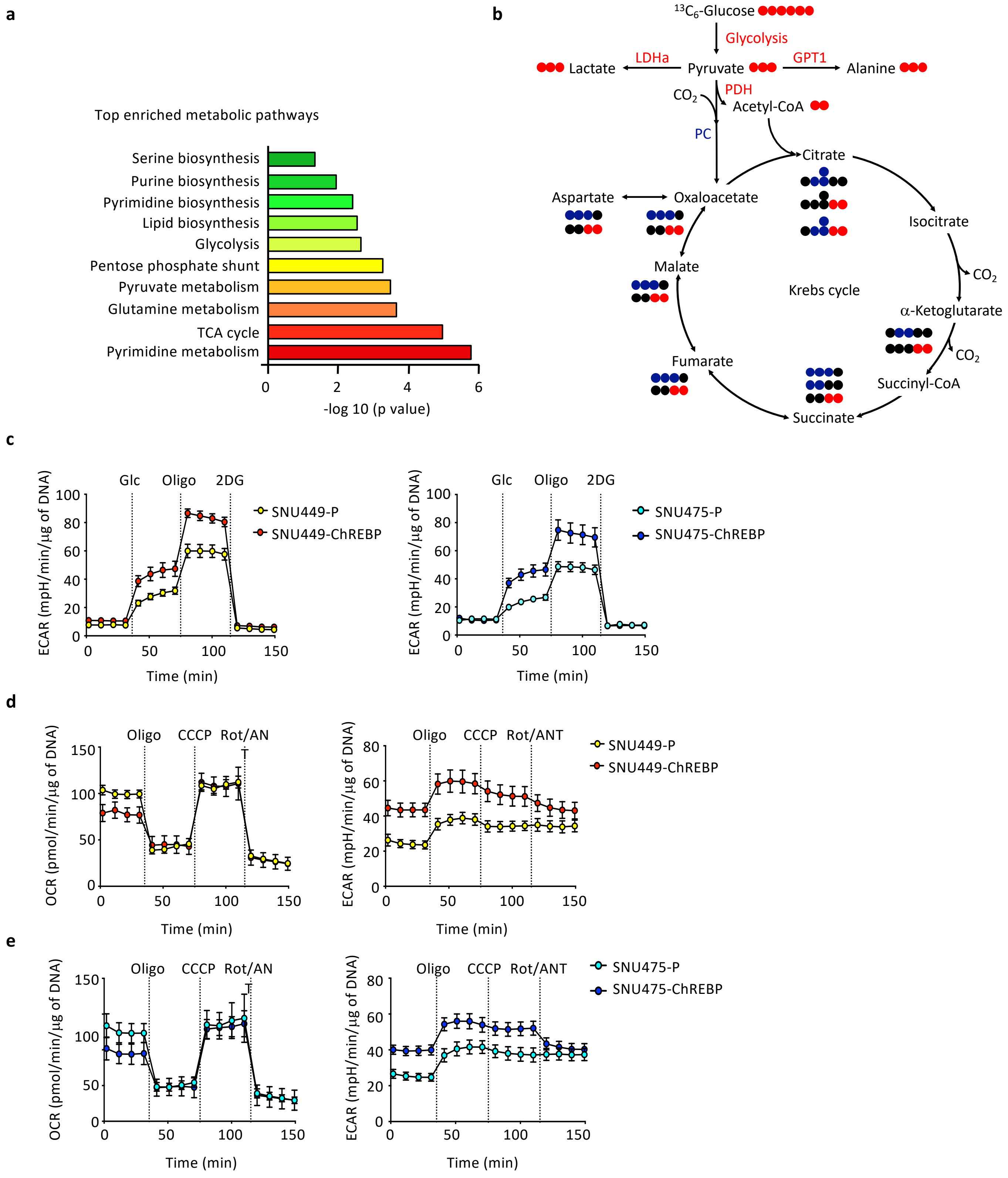
a
Top enriched metabolic pathways
Serine biosynthesis
Purine biosynthesis
Pyrimidine biosynthesis
Lipid biosynthesis
Glycolysis
Pentose phosphate shunt
Pyruvate metabolism
Glutamine metabolism
TCA cycle
Pyrimidine metabolism
-log 10 (p value)
b
13C6-Glucose
Glycolysis
LDHa
Lactate
Pyruvate
GPT1
Alanine
CO2
PDH
Acetyl-CoA
PC
Citrate
Aspartate
Oxaloacetate
Isocitrate
Malate
Krebs cycle
CO2
α-Ketoglutarate
CO2
Fumarate
Succinyl-CoA
Succinate
c
Glc
Oligo
2DG
ECAR (mpH/min/μg of DNA)
Time (min)
SNU449-P
SNU449-ChREBP
SNU475-P
SNU475-ChREBP
d
Oligo
CCCP
Rot/AN
Rot/ANT
OCR (pmol/min/μg of DNA)
ECAR (mpH/min/μg of DNA)
Time (min)
SNU449-P
SNU449-ChREBP
e
OCR (pmol/min/μg of DNA)
ECAR (mpH/min/μg of DNA)
Time (min)
SNU475-P
SNU475-ChREBP

Figure. S7

Supplementary Figure 7. Metabolic rewiring of glucose metabolism participates to ChREBP pro-proliferative effects. **(a)** Top ten enriched metabolic pathways from gene ontology analysis performed from ChREBP tumors (n = 12 biologically independent *mice* per group). **(b)** Schematic representation of $^{13}C_6$-glucose isotope tracing into glycolysis and TCA cycle via PDH or PC activity. **(c)** Representative profile after glycolysis stress assay showing the ECAR of parental or ChREBP overexpressing SNU449 (left) and SNU475 cells (right) (n = 9 independent experiments). **(d)** Representative profile after mitochondrial stress assay showing the OCR and ECAR of parental or ChREBP overexpressing SNU449 cells (n = 9 independent experiments). **(e)** Representative profile after mitochondrial stress assay showing the OCR and ECAR of parental or ChREBP overexpressing SNU475 cells (n = 9 independent experiments).

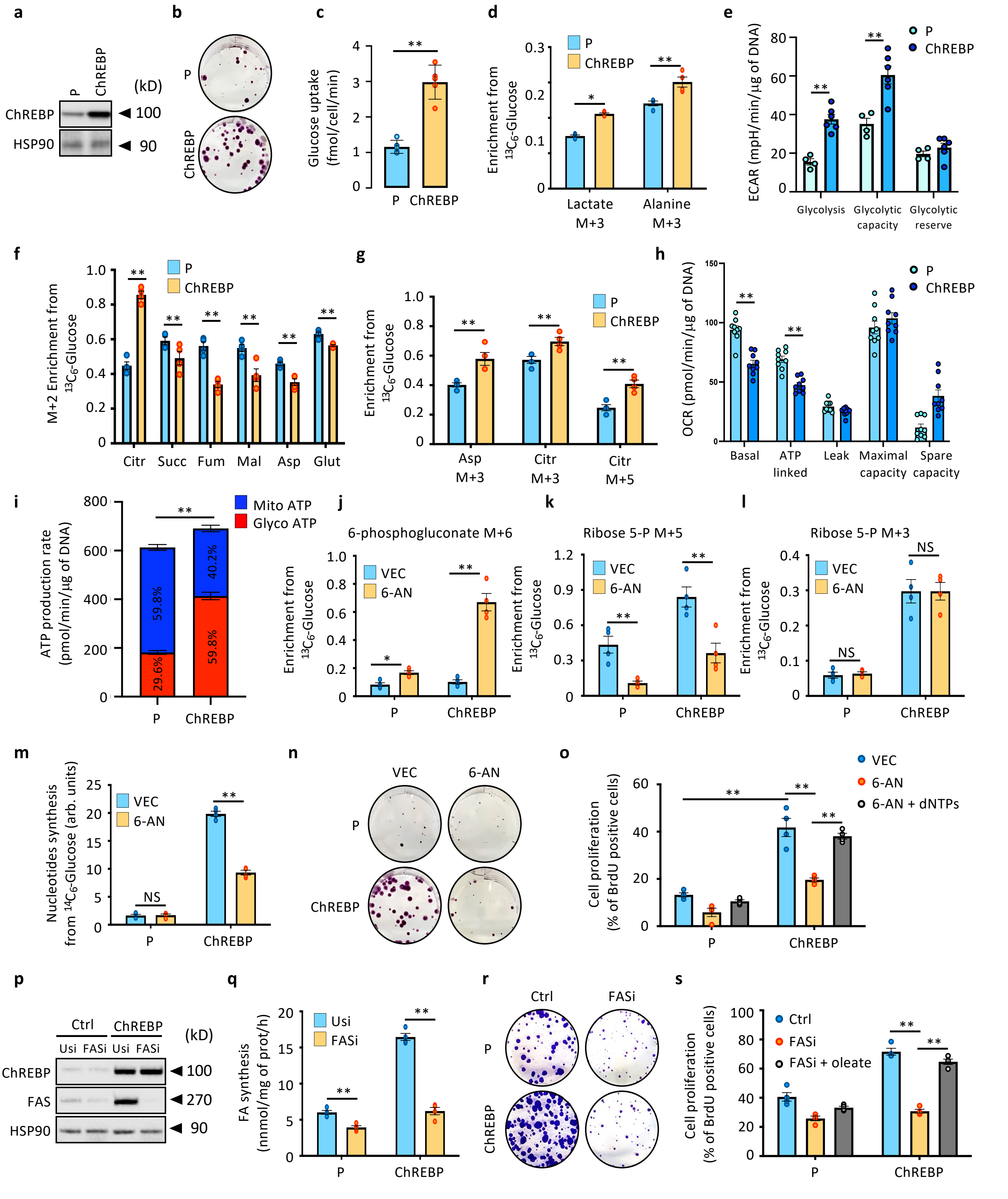
a
P
ChREBP
(kD)
ChREBP
HSP90
100
90
b
P
ChREBP
c
Glucose uptake (fmol/cell/min)
**
P
ChREBP
d
Enrichment from 13C6-Glucose
P
ChREBP
Lactate M+3
Alanine M+3
e
ECAR (mpH/min/µg of DNA)
Glycolysis
Glycolytic capacity
Glycolytic reserve
f
M+2 Enrichment from 13C6-Glucose
Citr
Succ
Fum
Mal
Asp
Glut
g
Enrichment from 13C6-Glucose
Asp M+3
Citr M+3
Citr M+5
h
OCR (pmol/min/µg of DNA)
Basal
ATP linked
Leak
Maximal capacity
Spare capacity
i
ATP production rate (pmol/min/µg of DNA)
Mito ATP
Glyco ATP
59.8%
29.6%
40.2%
59.8%
j
6-phosphogluconate M+6
VEC
6-AN
k
Ribose 5-P M+5
l
Ribose 5-P M+3
NS
m
Nucleotides synthesis from 14C6-Glucose (arb. units)
n
VEC
6-AN
o
Cell proliferation (% of BrdU positive cells)
6-AN + dNTPs
p
Ctrl
ChREBP
Usi
FASi
FAS
270
q
FA synthesis (nmol/mg of prot/h)
r
FASi
s
FASi + oleate

Figure. S8

Supplementary Figure 8. Metabolic rewiring of glucose metabolism in ChREBP overexpressing SNU475 cells. ChREBP was stably overexpressed using the Crispr/Cas9 technology in SNU475 hepatoma cell line. **(a)** Representative Western blot illustrating stable ChREBP overexpression. **(b)** Representative clonogenic assays shown (n = 3 independent experiments). **(c)** Rate of glucose uptake (n = 5 independent experiments). **(d)** Enrichment in (m+3) lactate and (m+3) alanine from $^{13}C_6$-glucose in parental or ChREBP overexpressing cells (n = 4 independent experiments). **(e)** Graph showing glycolysis, glycolytic capacity, and glycolytic reserve in parental or ChREBP overexpressing SNU475 (n = 9 independent experiments). Representative curve can be found in supplementary Figure 7c. **(f)** (m+2) enrichment in citrate (citr), succinate (succ), fumarate (fum), malate (mal), aspartate (asp) and glutamate (glut) from $^{13}C_6$-glucose in parental or ChREBP overexpressing SNU475 (n = 4 independent experiments). **(g)** Enrichment in (m+3) aspartate, (m+3) citrate and (m+5) citrate from $^{13}C_6$-glucose in parental or ChREBP overexpressing SNU475 (n = 4 independent experiments). **(h)** Graph showing basal OCR, proton leakage, maximal respiration, spare capacity, and ATP production of SNU475 overexpressing ChREBP (n = 9 independent experiments). Representative profile after mitochondrial stress assay showing the OCR of these SNU475 cells can be found in supplementary Figure 6e. **(i)** ATP production rate from glycolysis or oxidative phosphorylation from parental or ChREBP overexpressing SNU475 cells (n = 9 independent experiments). **(j-l)** Parental and ChREBP overexpressing SNU475 cells were treated with 6-AN (6-aminonicotinamide, 40 μM) for 24h. Cells were then incubated for 30 min with 11 mM of $^{13}C_6$-glucose. **(j)** Enrichment in (m+6) 6-phosphogluconate from $^{13}C_6$-glucose (n = 4 independent experiments). **(k)** Enrichment in (m+5) ribose 5-phopshate from $^{13}C_6$-glucose in response to ChREBP overexpression (n = 4 independent experiments). **(l)** Enrichment in (m+3) ribose 5-phopshate from $^{13}C_6$-glucose in parental or ChREBP overexpressing SNU475 (n = 4 independent experiments). **(m)** *De novo* nucleotide synthesis from parental and ChREBP overexpressing SNU475 cells incubated 6h with 11 mM of $^{14}C_6$-labelled glucose (n = 4 independent experiments). **(n)** Effect of 6-AN treatment on ChREBP-mediated increase in hepatocyte proliferation was studied in SNU475 cells. Representative clonogenic assays shown (n = 7 independent experiments). **(o)** Effect of 6-AN treatment (40 μM, 24h) and dNTPs rescue (100 μM each) on ChREBP-mediated increase in cell proliferation. Cell proliferation index was determined by measuring the % of BrdU positive SNU475 cells (n = 4 independent experiments). **(p-s)** FAS expression was knockdown in ChREBP overexpressing SNU449 cells by Crispr/Cas9. **(p)** Representative Western blot showing FAS deletion in ChREBP overexpressing SNU475 (n = 3 independent experiments). **(q)** *De novo* lipid synthesis from $^{14}C_6$-labelled glucose. SNU475 cells were incubated 6h with 11 mM of $^{14}C_6$-labelled glucose (n = 3 independent experiments). **(r)** Representative clonogenic assays shown after FAS deletion (n = 3 independent experiments). **(s)** Effect of FAS knockdown and oleate supplementation (50 μM) on ChREBP-mediated increase in cell proliferation. Cell proliferation index was determined by measuring the % of BrdU positive cells (n = 4 independent experiments). All error bars represent mean ± SEM. (c, d, e, f, g, h, i) Statistical analyses were made using unpaired two-sided Student's t-test * $P < 0.05$. **$P < 0.001$. (j, k, l, m, o, q, s) Statistical analyses were made using two-way ANOVA and Tukey's multiple-comparisons test. * $P < 0.05$. ** $P < 0.001$. NS = non-significant.

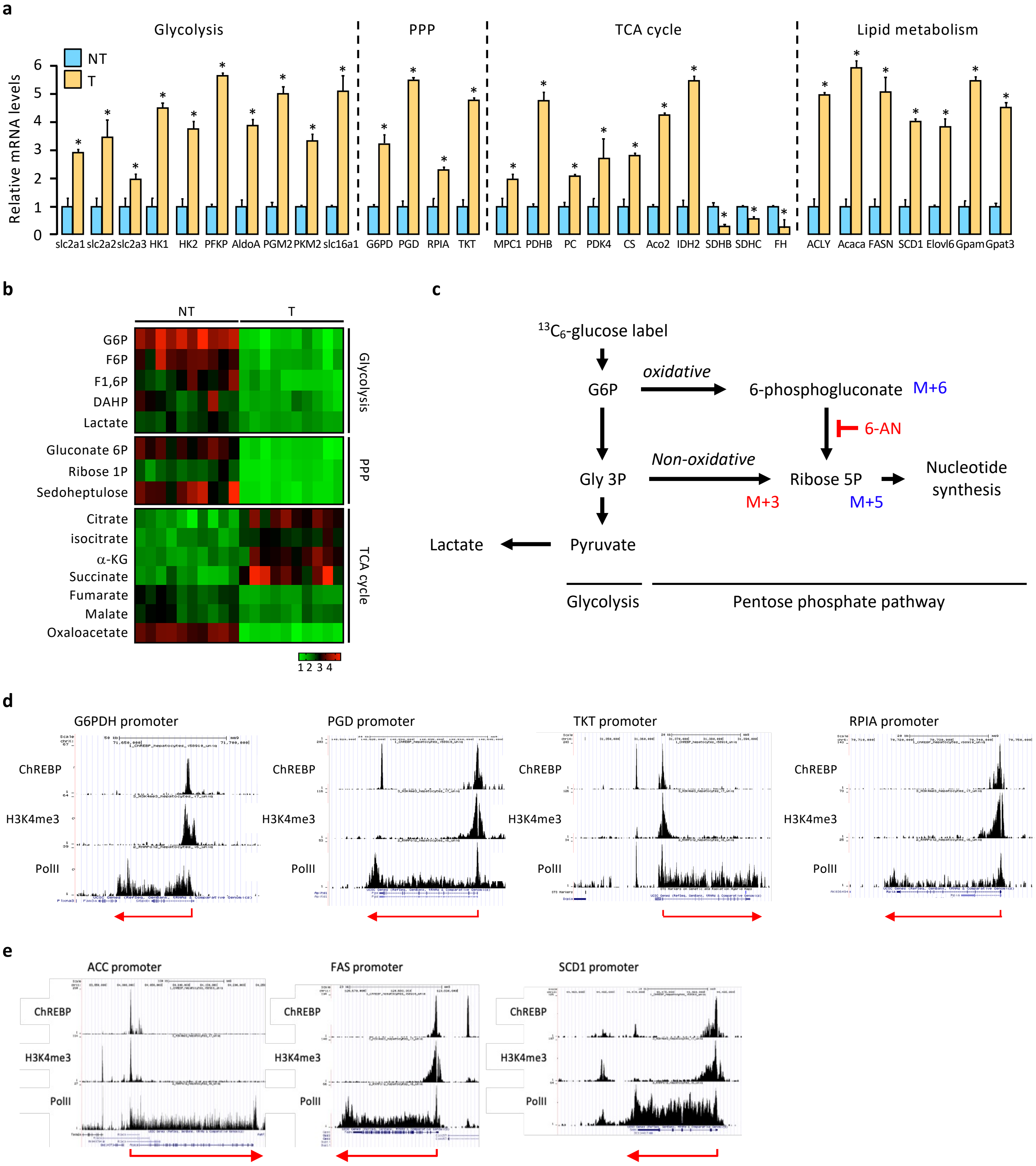
a
Glycolysis
PPP
TCA cycle
Lipid metabolism
NT
T
Relative mRNA levels
slc2a1 slc2a2 slc2a3 HK1 HK2 PFKP AldoA PGM2 PKM2 slc16a1 G6PD PGD RPIA TKT MPC1 PDHB PC PDK4 CS Aco2 IDH2 SDHB SDHC FH ACLY Acaca FASN SCD1 Elovl6 Gpam Gpat3
b
NT
T
G6P
F6P
F1,6P
DAHP
Lactate
Glycolysis
Gluconate 6P
Ribose 1P
Sedoheptulose
PPP
Citrate
isocitrate
α-KG
Succinate
Fumarate
Malate
Oxaloacetate
TCA cycle
1 2 3 4
c
13C6-glucose label
G6P
oxidative
6-phosphogluconate M+6
6-AN
Gly 3P
Non-oxidative
Ribose 5P
Nucleotide synthesis
M+3
M+5
Lactate
Pyruvate
Glycolysis
Pentose phosphate pathway
d
G6PDH promoter
PGD promoter
TKT promoter
RPIA promoter
ChREBP
H3K4me3
PolII
e
ACC promoter
FAS promoter
SCD1 promoter
ChREBP
H3K4me3
PolII

Figure. S9

Supplementary Figure 9. Metabolic rewiring of glucose metabolism in ChREBP overexpressing tumors. **(a)** Relative expression of glycolytic, PPP, TCA cycle and lipogenic genes in ChREBP tumors. The expression of a specific gene was normalized to TBP mRNA levels (n = 12 biologically independent *mice* per group). **(b)** Heatmap of glycolytic, PPP and TCA cycle metabolic intermediates between NT and T samples (n = 10 biologically independent *mice* per group). **(c)** Schematic representation of $^{13}C_6$-glucose isotope tracing into the pentose phosphate shunt. **(d)** UCSC genome browser image illustrating normalized tag counts for ChREBP, H3K4me3 and RNA polII at the promoter of G6PDH, PGD, TKT and RPIA. **(e)** UCSC genome browser image illustrating normalized tag counts for ChREBP, H3K4me3 and RNA polII at the promoter of lipogenic genes. All error bars represent mean ± SEM. (a) Statistical analyses were made using unpaired two-sided Student's t-test. * $P < 0.01$.

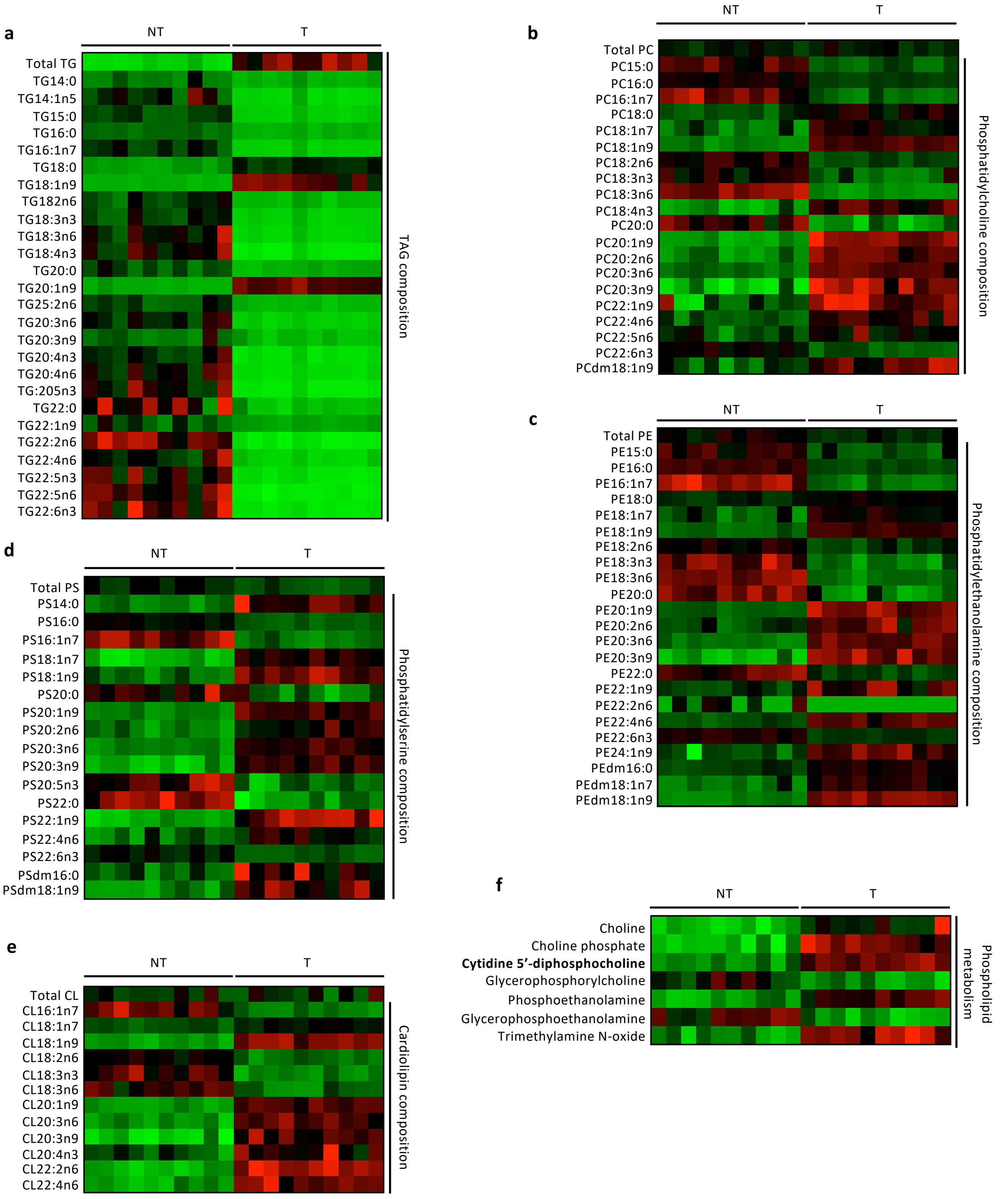
a
NT
T
Total TG
TG14:0
TG14:1n5
TG15:0
TG16:0
TG16:1n7
TG18:0
TG18:1n9
TG182n6
TG18:3n3
TG18:3n6
TG18:4n3
TG20:0
TG20:1n9
TG25:2n6
TG20:3n6
TG20:3n9
TG20:4n3
TG20:4n6
TG:205n3
TG22:0
TG22:1n9
TG22:2n6
TG22:4n6
TG22:5n3
TG22:5n6
TG22:6n3
TAG composition
b
NT
T
Total PC
PC15:0
PC16:0
PC16:1n7
PC18:0
PC18:1n7
PC18:1n9
PC18:2n6
PC18:3n3
PC18:3n6
PC18:4n3
PC20:0
PC20:1n9
PC20:2n6
PC20:3n6
PC20:3n9
PC22:1n9
PC22:4n6
PC22:5n6
PC22:6n3
PCdm18:1n9
Phosphatidylcholine composition
c
NT
T
Total PE
PE15:0
PE16:0
PE16:1n7
PE18:0
PE18:1n7
PE18:1n9
PE18:2n6
PE18:3n3
PE18:3n6
PE20:0
PE20:1n9
PE20:2n6
PE20:3n6
PE20:3n9
PE22:0
PE22:1n9
PE22:2n6
PE22:4n6
PE22:6n3
PE24:1n9
PEdm16:0
PEdm18:1n7
PEdm18:1n9
Phosphatidylethanolamine composition
d
NT
T
Total PS
PS14:0
PS16:0
PS16:1n7
PS18:1n7
PS18:1n9
PS20:0
PS20:1n9
PS20:2n6
PS20:3n6
PS20:3n9
PS20:5n3
PS22:0
PS22:1n9
PS22:4n6
PS22:6n3
PSdm16:0
PSdm18:1n9
Phosphatidylserine composition
e
NT
T
Total CL
CL16:1n7
CL18:1n7
CL18:1n9
CL18:2n6
CL18:3n3
CL18:3n6
CL20:1n9
CL20:3n6
CL20:3n9
CL20:4n3
CL22:2n6
CL22:4n6
Cardiolipin composition
f
NT
T
Choline
Choline phosphate
Cytidine 5'-diphosphocholine
Glycerophosphorylcholine
Phosphoethanolamine
Glycerophosphoethanolamine
Trimethylamine N-oxide
Phospholipid metabolism

Figure. S10

Supplementary Figure 10. (a-e) Heatmap visualization of relative SFA, MUFA, and PUFA content in triglycrides (TG) **(a)** and phospholipid species including phophatidylcholine (PC) **(b)**, phosphatidylethanolamine (PE) **(c)**, phosphatidylserine (PS) **(d)** and cardiolipin (CL) **(e)** in ChREBP tumors (T) compared to non-tumoral tissue (NT). Total content of each lipid species is indicated in the first line of each heatmap (n = 15 biologically independent *mice* per group). **(f)** Heatmap of metabolic intermediates of phospholipid metabolism between NT and T samples (n = 15 biologically independent *mice* per group).

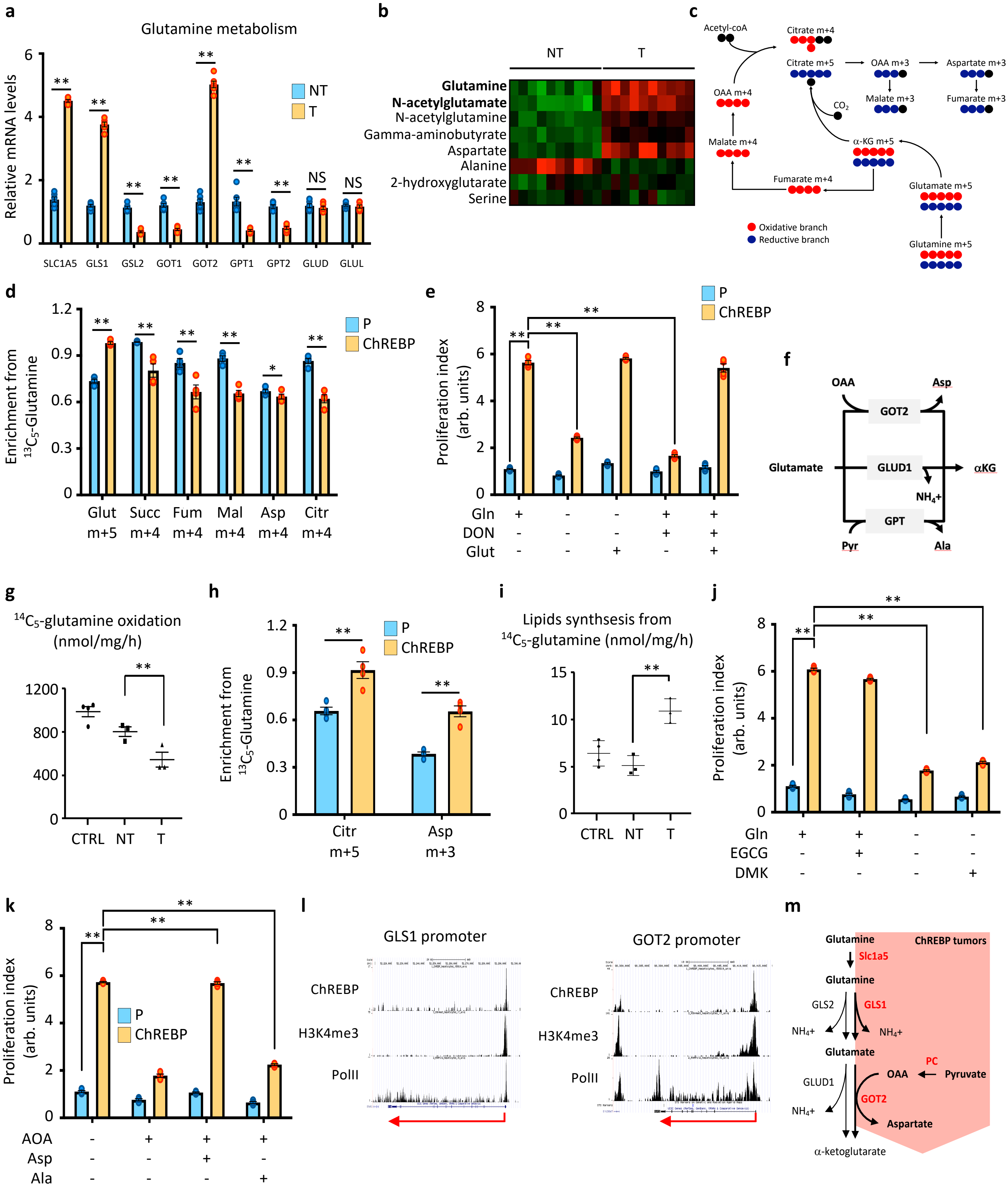

a
Glutamine metabolism
Relative mRNA levels
NT
T
SLC1A5
GLS1
GSL2
GOT1
GOT2
GPT1
GPT2
GLUD
GLUL
NS
b
NT
T
Glutamine
N-acetylglutamate
N-acetylglutamine
Gamma-aminobutyrate
Aspartate
Alanine
2-hydroxyglutarate
Serine
c
Acetyl-coA
Citrate m+4
Citrate m+5
OAA m+3
Aspartate m+3
OAA m+4
Malate m+3
Fumarate m+3
CO_2
Malate m+4
α-KG m+5
Fumarate m+4
Glutamate m+5
Glutamine m+5
Oxidative branch
Reductive branch
d
Enrichment from $^{13}C_5$-Glutamine
P
ChREBP
Glut m+5
Succ m+4
Fum m+4
Mal m+4
Asp m+4
Citr m+4
e
Proliferation index (arb. units)
Gln
DON
Glut
f
OAA
Asp
GOT2
Glutamate
GLUD1
αKG
NH_4+
GPT
Pyr
Ala
g
$^{14}C_5$-glutamine oxidation (nmol/mg/h)
CTRL
NT
T
h
Citr m+5
Asp m+3
i
Lipids synthsesis from $^{14}C_5$-glutamine (nmol/mg/h)
j
EGCG
DMK
k
AOA
Asp
Ala
l
GLS1 promoter
GOT2 promoter
ChREBP
H3K4me3
PolII
m
ChREBP tumors
Glutamine
Slc1a5
GLS2
GLS1
NH_4+
Glutamate
PC
GLUD1
OAA
Pyruvate
GOT2
Aspartate
α-ketoglutarate

Figure. S11

Supplementary Figure 11. Metabolic rewiring of glutamine metabolism in response to ChREBP overexpression. **(a)** Relative expression of genes involved in glutamine metabolism in ChREBP tumors. The expression of a specific gene is normalized to TBP mRNA levels (n = 12 biologically independent *mice* per group). **(b)** Heatmap of metabolic intermediates of Gln metabolism in ChREBP tumors (n = 10 biologically independent *mice* per group). **(c)** Schematic representation of $^{13}C_5$-glutamine isotope tracing into the TCA cycle via oxidative or reductive metabolism. **(d)** (m+5) enrichment in glutamate, (m+4) succinate, (m+4) fumarate, (m+4) malate, (m+4) aspartate and (m+4) citrate from $^{13}C_5$-glutamine in parental or ChREBP overexpressing SNU475 cells (n = 4 independent experiments). **(e)** Proliferation index of SNU475 cells overexpressing ChREBP after Gln deprivation or GLS inhibition (n = 4 independent experiments). Glutamate (Glu) 4 mM. **(f)** Schematic representation of mitochondrial Glut metabolism involving GLUD1, GOT2 and GPT2. **(g)** Gln oxidation rate determined by measuring the production of $^{14}CO_2$ from $^{14}C_5$-Gln for 4h (n = 6 biologically independent *mice* per group). **(h)** Enrichment in (m+3) citrate and (m+3) aspartate from $^{13}C_5$-glutamine in parental or ChREBP overexpressing SNU475 cells (n = 4 independent experiments). **(i)** Measurement of *de novo* lipid synthesis from $^{14}C_5$-labelled Gln in ChREBP tumors (n = 3 biologically independent *mice* per group). **(j)** Proliferation index of SNU475 cells overexpressing ChREBP after GLUD1 inhibition (n = 4 independent experiments). **(k)** Proliferation index of SNU475 cells overexpressing ChREBP after AOA treatment (n = 4 independent experiments). **(l)** UCSC genome browser image illustrating normalized tag counts for ChREBP, H3K4me3 and RNA polII at the promoter of GLS1 and GOT2. **(m)** Schematic representation of Gln metabolic rerouting in response to ChREBP activation. All error bars represent mean ± SEM. (a, d, h) Statistical analyses were made using unpaired two-sided Student's t-test. * $P < 0.05$. ** $P < 0.01$. (e, g, i, j, k) Statistical analyses were made using two-way ANOVA and Tukey's multiple-comparisons test. ** $P < 0.001$.

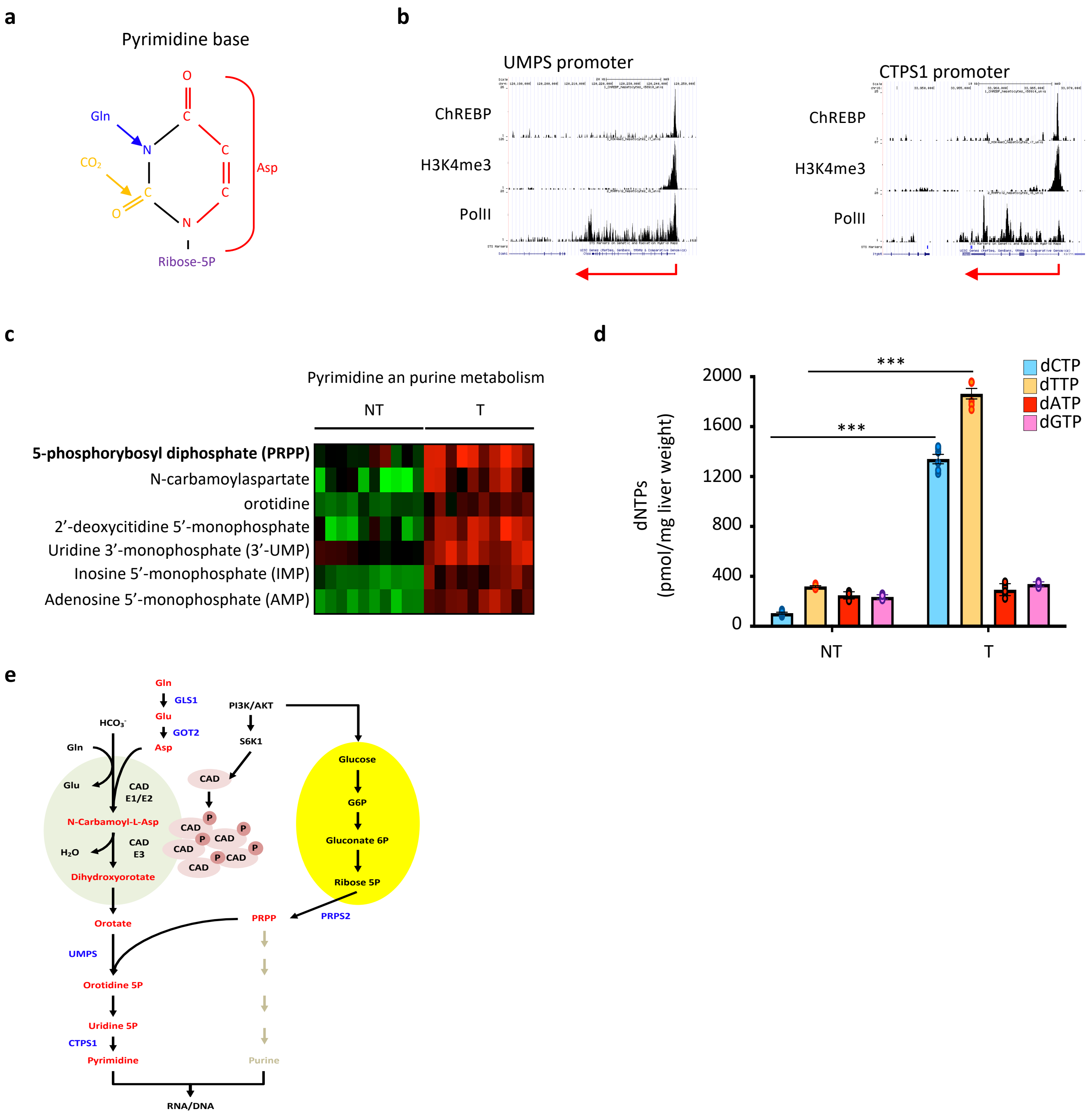

a
Pyrimidine base
O
C
Gln
N
C
CO2
C
Asp
C
O
N
Ribose-5P
b
UMPS promoter
ChREBP
H3K4me3
PolII
CTPS1 promoter
ChREBP
H3K4me3
PolII
c
Pyrimidine an purine metabolism
NT
T
5-phosphorybosyl diphosphate (PRPP)
N-carbamoylaspartate
orotidine
2'-deoxycitidine 5'-monophosphate
Uridine 3'-monophosphate (3'-UMP)
Inosine 5'-monophosphate (IMP)
Adenosine 5'-monophosphate (AMP)
d
dNTPs (pmol/mg liver weight)
2000
1600
1200
800
400
0
***
***
dCTP
dTTP
dATP
dGTP
NT
T
e
Gln
GLS1
Glu
GOT2
Asp
HCO3-
Gln
Glu
CAD E1/E2
N-Carbamoyl-L-Asp
H2O
CAD E3
Dihydroxyorotate
PI3K/AKT
S6K1
CAD
P
CAD
Glucose
G6P
Gluconate 6P
Ribose 5P
PRPP
PRPS2
Orotate
UMPS
Orotidine 5P
Uridine 5P
CTPS1
Pyrimidine
Purine
RNA/DNA

Figure. S12

Supplementary Figure 12. ChREBP favors tumor growth by channeling glutamine metabolism into *de novo* pyrimidine synthesis. **(a)** Sketch illustrating how Gln, Asp and glucose are used in the process of *de novo* pyrimidine synthesis, Asp being the carbon donor. **(b)** UCSC genome browser image illustrating normalized tag counts for ChREBP, H3K4me3 and RNA polII at the promoter of UMPS and CTPS1. **(c)** Heatmap of metabolic intermediates of pyrimidine synthesis in ChREBP overexpressing tumors (n = 10 biologically independent *mice* per group). **(d)** dNTP pools measured by HPLC in ChREBP overexpressing tumors (n = 6 biologically independent *mice* per group) **(e)** Illustration of the role of ChREBP in controlling glucose, Gln and Asp metabolism to support *de novo* pyrimidine synthesis. All error bars represent mean ± SEM. (d) Statistical analyses were made using two-way ANOVA and Tukey's multiple-comparisons test. *** $P < 0.001$.

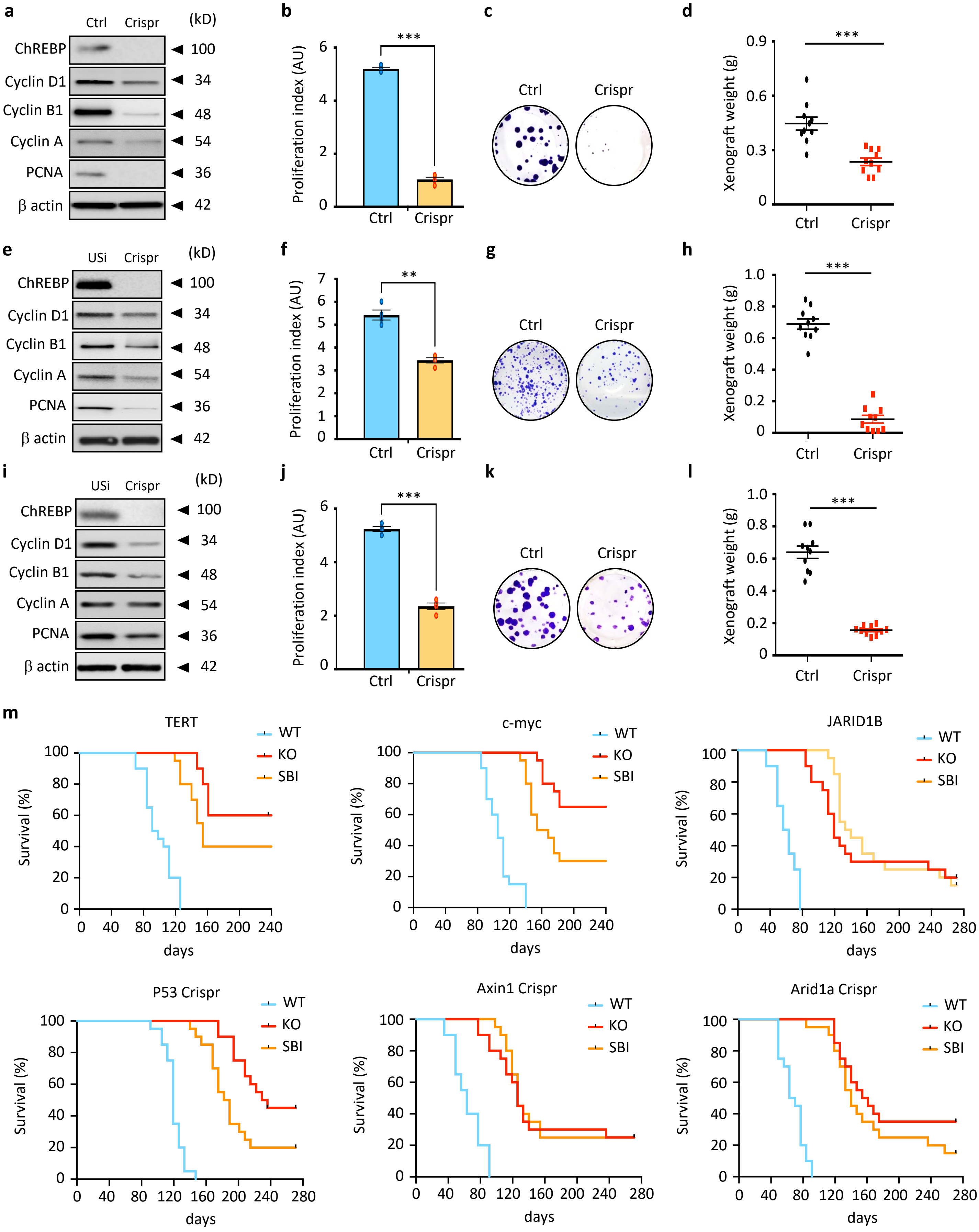

a
Ctrl Crispr (kD)
ChREBP 100
Cyclin D1 34
Cyclin B1 48
Cyclin A 54
PCNA 36
β actin 42
b
Proliferation index (AU)
***
Ctrl Crispr
c
Ctrl Crispr
d
Xenograft weight (g)
***
Ctrl Crispr
e
USi Crispr (kD)
ChREBP 100
Cyclin D1 34
Cyclin B1 48
Cyclin A 54
PCNA 36
β actin 42
f
Proliferation index (AU)
**
Ctrl Crispr
g
Ctrl Crispr
h
Xenograft weight (g)
***
Ctrl Crispr
i
USi Crispr (kD)
ChREBP 100
Cyclin D1 34
Cyclin B1 48
Cyclin A 54
PCNA 36
β actin 42
j
Proliferation index (AU)
***
Ctrl Crispr
k
Ctrl Crispr
l
Xenograft weight (g)
***
Ctrl Crispr
m
TERT
c-myc
JARID1B
P53 Crispr
Axin1 Crispr
Arid1a Crispr
WT
KO
SBI
Survival (%)
days

Figure. S13

Supplementary Figure 13. ChREBP represents a promising target for HCC treatment. **(a-l)** ChREBP expression was stably inhibited in SNU475, Huh7 and Bnl cl.2 cells using the Crispr/Cas9 approach. **(a, e and i)** Representative Western blot analysis of cell cycle proteins in SNU475 (a), Huh7 (e) and Bnl cl.2 (i) deficient for ChREBP (n = 3 independent experiments). **(b, f and j)** Proliferation index of ChREBP deficient SNU475 (b), Huh7 (f) and Bnl cl.2 (j) cells (n = 4 independent experiments). **(c, g and k)** Representative clonogenic assays from ChREBP deficient SNU475 (c), Huh7 (g) and Bnl cl.2 (k) cells (n = 7 independent experiments). **(d, h and l)** Xenograft mouse model of HCC established in nude *mice* using parental and ChREBP deficient SNU475 (d), Huh7 (h) and Bnl cl.2 (l) cells. Representative tumor weight shown at 3 weeks for each cell types (n = 10 biologically independent *mice* per group). **(m)** Kaplan–Meier analysis depicting *mice* overall survival rate in response to TERT, c-myc and JARID1B overexpression or in response to p53, axin1 and arid1a knockdown in liver-specific ChREBP KO mice or in response to SBI-993 treatment. Number of *mice* at risk at the specific time point can be found in the Source data file. All error bars represent mean ± SEM. (b, d, f, h, j, l) Statistical analyses were made using unpaired two-sided Student's t-test. *** $P < 0.001$. (m) Significant difference in survival between cohorts was calculated using the log-rank (Mantel Cox) test.

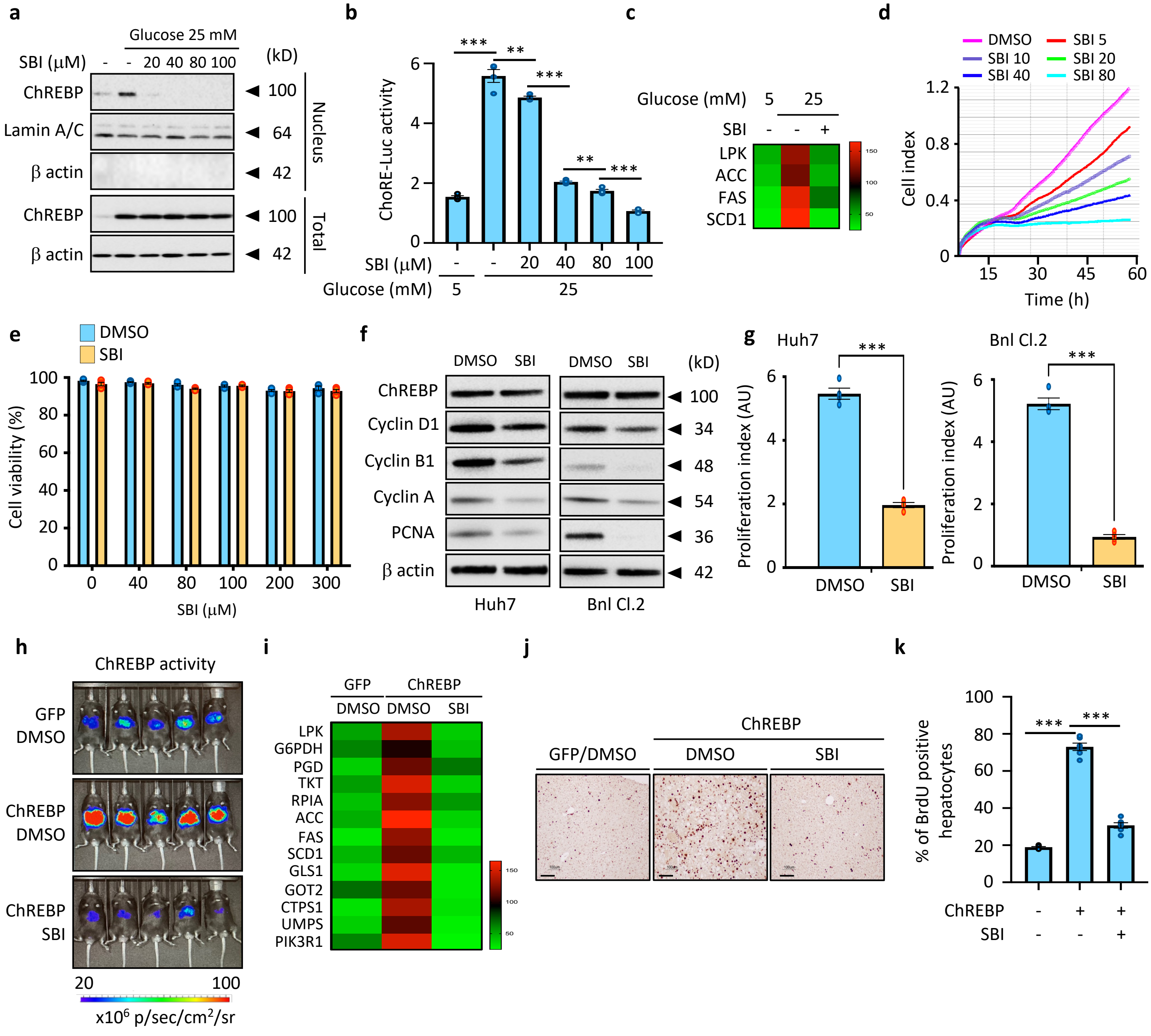
a
Glucose 25 mM
SBI (μM) - - 20 40 80 100
(kD)
ChREBP
Lamin A/C
β actin
ChREBP
β actin
100
64
42
100
42
Nucleus
Total
b
ChoRE-Luc activity
SBI (μM) - - 20 40 80 100
Glucose (mM) 5 25
c
Glucose (mM) 5 25
SBI - - +
LPK
ACC
FAS
SCD1
d
DMSO
SBI 5
SBI 10
SBI 20
SBI 40
SBI 80
Cell index
Time (h)
e
DMSO
SBI
Cell viability (%)
SBI (μM)
f
DMSO SBI DMSO SBI (kD)
ChREBP
Cyclin D1
Cyclin B1
Cyclin A
PCNA
β actin
100
34
48
54
36
42
Huh7
Bnl Cl.2
g
Huh7
Bnl Cl.2
Proliferation index (AU)
DMSO SBI
h
ChREBP activity
GFP DMSO
ChREBP DMSO
ChREBP SBI
20 100
x10^6 p/sec/cm^2/sr
i
GFP ChREBP
DMSO DMSO SBI
LPK
G6PDH
PGD
TKT
RPIA
ACC
FAS
SCD1
GLS1
GOT2
CTPS1
UMPS
PIK3R1
j
ChREBP
GFP/DMSO DMSO SBI
k
% of BrdU positive hepatocytes
ChREBP - + +
SBI - - +

Figure. S14

Supplementary Figure 14. SBI-993 treatment significantly inhibits ChREBP activity and cell proliferation. **(a)** Representative Western blot analyzing the effect of SBI-993 on ChREBP nuclear translocation in primary cultured hepatocytes (n = 3 independent experiments). **(b)** Measurement of ChREBP transcriptional activity in primary cultured hepatocytes in response to SBI-993 treatment (n = 3 independent experiments). **(c)** Expression of ChREBP-regulated genes in response to glucose (25 mM) stimulation and SBI-993 (40 μM) treatment in primary cultured hepatocytes (n = 3 independent experiments). **(d)** Proliferation index of parental SNU449 cells in response to SBI-993 treatment (n = 3 independent experiments). **(e)** Effect of SBI-993 on SNU449 cell viability measured using CCK8 assay (n = 3 independent experiments). **(f)** Representative Western blot analysis of cell cycle proteins in parental Huh7 and Bnl Cl.2 cells treated with SBI-993 (40 μM) (n = 3 independent experiments). **(g)** Proliferation index of parental Huh7 and Bnl Cl.2 cells treated with SBI-993 (40 μM) (n = 4 independent experiments). **(h-k)** C57Bl6/J male *mice*, infected with either GFP or ChREBP overexpressing adenovirus, were daily IP injected with SBI-993 (50 mg/kg) for 3 consecutive weeks. **(h)** Representative image of ChREBP transcriptional activity on Chore-luc reporter construct *in vivo* (n = 6 biologically independent *mice* per group). **(i)** Expression of ChREBP-regulated genes relative to TBP expression from mice treated with SBI-993 in the form of an heatmap (n = 6 biologically independent *mice* per group). **(j)** Representative staining of liver sections with BrdU (n = 6 biologically independent *mice* per group). Scale bars = 100 μm. **(k)** Quantification of the percentage of BrdU positive hepatocytes from the BrdU staining (n = 6 biologically independent *mice* per group). All error bars represent mean ± SEM. (g) Statistical analyses were made using unpaired two-sided Student's t-test. *** $P < 0.001$. (b, e, k) Statistical analyses were made using two-way ANOVA and Tukey's multiple-comparisons test. ** $P < 0.01$. *** $P < 0.001$.

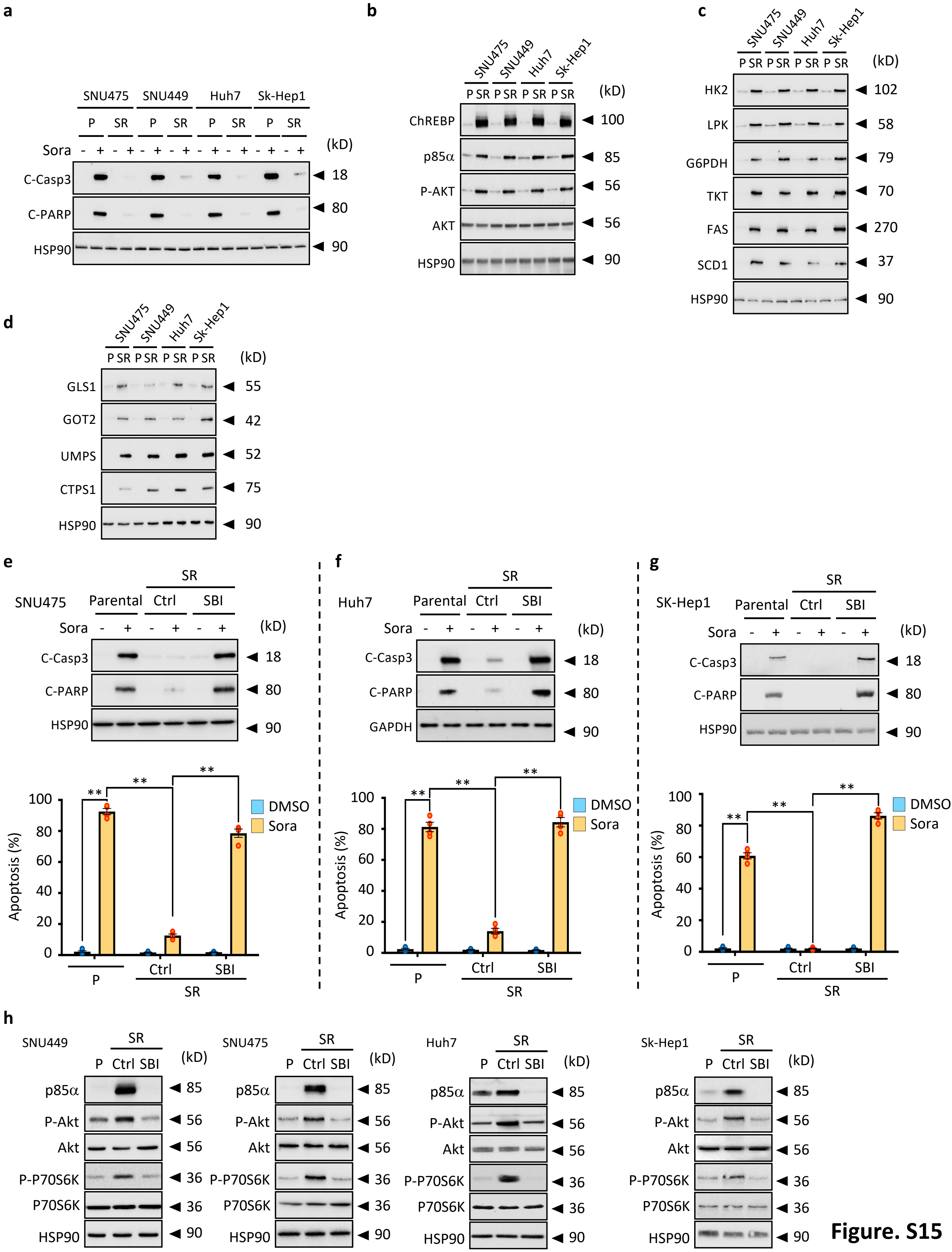
a
SNU475 SNU449 Huh7 Sk-Hep1
P SR P SR P SR P SR
Sora - + - + - + - + - + - + - + - +
(kD)
C-Casp3 18
C-PARP 80
HSP90 90
b
SNU475 SNU449 Huh7 Sk-Hep1
P SR P SR P SR P SR (kD)
ChREBP 100
p85α 85
P-AKT 56
AKT 56
HSP90 90
c
SNU475 SNU449 Huh7 Sk-Hep1
P SR P SR P SR P SR (kD)
HK2 102
LPK 58
G6PDH 79
TKT 70
FAS 270
SCD1 37
HSP90 90
d
SNU475 SNU449 Huh7 Sk-Hep1
P SR P SR P SR P SR (kD)
GLS1 55
GOT2 42
UMPS 52
CTPS1 75
HSP90 90
e
SNU475 Parental SR Ctrl SBI
Sora - + - + - + (kD)
C-Casp3 18
C-PARP 80
HSP90 90
Apoptosis (%)
DMSO
Sora
P Ctrl SBI SR
f
Huh7 Parental SR Ctrl SBI
Sora - + - + - + (kD)
C-Casp3 18
C-PARP 80
GAPDH 90
Apoptosis (%)
DMSO
Sora
P Ctrl SBI SR
g
SK-Hep1 Parental SR Ctrl SBI
Sora - + - + - + (kD)
C-Casp3 18
C-PARP 80
HSP90 90
Apoptosis (%)
DMSO
Sora
P Ctrl SBI SR
h
SNU449 SNU475 Huh7 Sk-Hep1
P SR Ctrl SBI (kD)
p85α 85
P-Akt 56
Akt 56
P-P70S6K 36
P70S6K 36
HSP90 90

Figure. S15

Supplementary Figure 15. SBI-993 reverses sorafenib resistance. **(a)** Representative Western blot showing caspase 3 and PARP activity in response to sorafenib treatment (6h, 15 μM) on parental or sorafenib resistant SNU475, SNU449, Huh7, and SK-Hep1 cells (n = 3 independent experiments). P (parental), SR (sorafenib resistant clone) **(b)** Representative Western blot of the expression of ChREBP and the activity of the PI3K/AKT signaling pathway in parental or sorafenib resistant SNU475, SNU449, Huh7, and SK-Hep1 cells (n = 3 independent experiments). **(c and d)** Representative Western blot depicting glucose and glutamine metabolic reprogramming in parental or sorafenib resistant SNU475, SNU449, Huh7, and SK-Hep1 cells (n = 3 independent experiments). **(e-g)** Effect of SBI-993 and sorafenib co-treatment on SR-SNU475 (left panel), SR-Huh7 (middle panel) and SK-Hep1 (right panel) cell apoptosis. (Top panel) Representative Western blot depicting caspase 3 and PARP activity in indicated cell lines treated with sorafenib alone or in combination with SBI-993 (n = 3 independent experiments). (Bottom panel) Measurement of cell apoptosis (n = 4 independent experiments). **(h)** Representative Western blot depicting the effect of SBI-993 on PI3K/AKT signaling in SR-SNU449, SR-SNU475, SR-Huh7 and SR-SK-Hep1 cells (n = 3 independent experiments). All error bars represent mean ± SEM. Statistical analyses were made using two-way ANOVA and Tukey's multiple-comparisons test. ** $P < 0.01$.

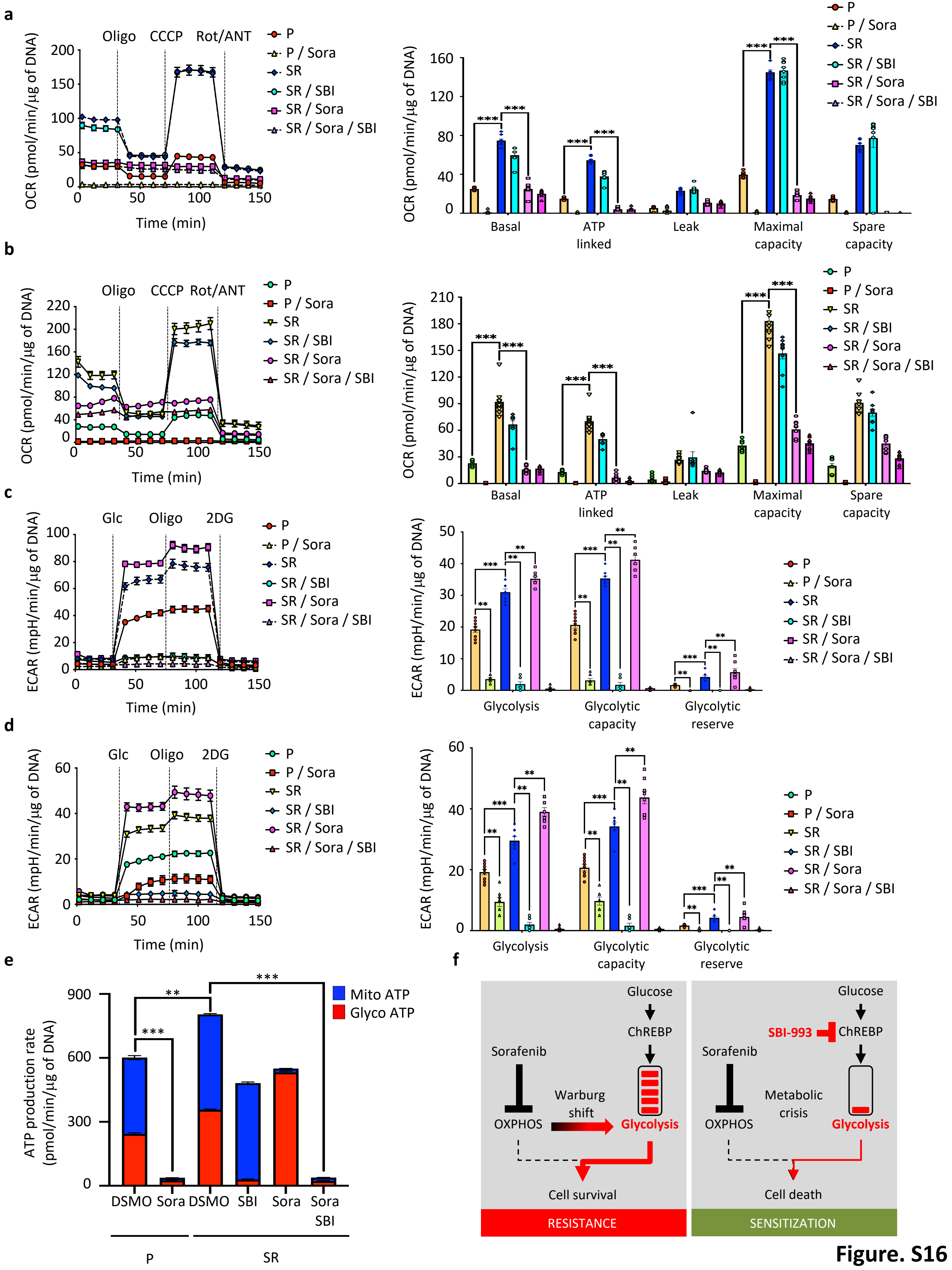

a
Oligo
CCCP
Rot/ANT
OCR (pmol/min/µg of DNA)
Time (min)
P
P / Sora
SR
SR / SBI
SR / Sora
SR / Sora / SBI
Basal
ATP linked
Leak
Maximal capacity
Spare capacity
b
Oligo
CCCP
Rot/ANT
OCR (pmol/min/µg of DNA)
Time (min)
c
Glc
Oligo
2DG
ECAR (mpH/min/µg of DNA)
Time (min)
Glycolysis
Glycolytic capacity
Glycolytic reserve
d
Glc
Oligo
2DG
ECAR (mpH/min/µg of DNA)
Time (min)
e
ATP production rate (pmol/min/µg of DNA)
Mito ATP
Glyco ATP
DSMO
Sora
DSMO
SBI
Sora
Sora SBI
P
SR
f
Glucose
ChREBP
Sorafenib
Warburg shift
OXPHOS
Glycolysis
Cell survival
RESISTANCE
Glucose
SBI-993
ChREBP
Sorafenib
Metabolic crisis
OXPHOS
Glycolysis
Cell death
SENSITIZATION

Figure. S16

Supplementary Figure 16. SBI-993 reverses sorafenib resistance by preventing metabolic flexibility upon sorafenib treatment. Parental and sorafenib resistant SNU449 and SNU475 cells were incubated 6h with sorafenib (15 μM) prior to the characterization of their mitochondrial and glycolytic functions. SR-SNU449 and SR-SNU475 were pre-incubated or not with SBI-993 (40 μM) for 24h before sorafenib treatment. **(a)** (left panel) Representative profile after mitochondrial stress assay showing the OCR of parental or sorafenib resistant SNU449 cells in response to sorafenib and/or SBI-993 treatment (n = 9 independent experiments). (Right panel) Graph showing basal OCR, proton leakage, maximal respiration, spare capacity, and ATP production (n = 9 independent experiments). **(b)** (left panel) Representative profile after mitochondrial stress assay showing the OCR of parental or sorafenib resistant SNU475 cells in response to sorafenib and/or SBI-993 treatment (n = 9 independent experiments). (Right panel) Graph showing basal OCR, proton leakage, maximal respiration, spare capacity, and ATP production (n = 9 independent experiments). **(c)** (left panel) Representative profile after glycolysis stress test showing the ECAR of parental or sorafenib resistant SNU449 cells in response to sorafenib and/or SBI-993 treatment (n = 9 independent experiments). (Right panel) Graph showing basal glycolysis, glycolytic capacity, and glycolytic reserve (n = 9 independent experiments). **(d)** (left panel) Representative profile after glycolysis stress test showing the ECAR of parental or sorafenib resistant SNU475 cells in response to sorafenib and/or SBI-993 treatment (n = 9 independent experiments). (Right panel) Graph showing basal glycolysis, glycolytic capacity, and glycolytic reserve (n = 9 independent experiments). **(e)** ATP production rate from glycolysis or oxidative phosphorylation in parental or sorafenib resistant SNU475 cells (n = 9 independent experiments). **(f)** In resistant HCC models, sorafenib works noncanonically to inhibit OXPHOS, leading to a shift to glycolysis and survival. However, pharmacological inhibition of ChREBP quenches glycolytic flux and sensitizes HCC cells to sorafenib action. All error bars represent mean ± SEM. (a, b, c, d, e) Statistical analyses were made using two-way ANOVA and Tukey’s multiple-comparisons test. ** $P < 0.01$. *** $P < 0.001$.

a

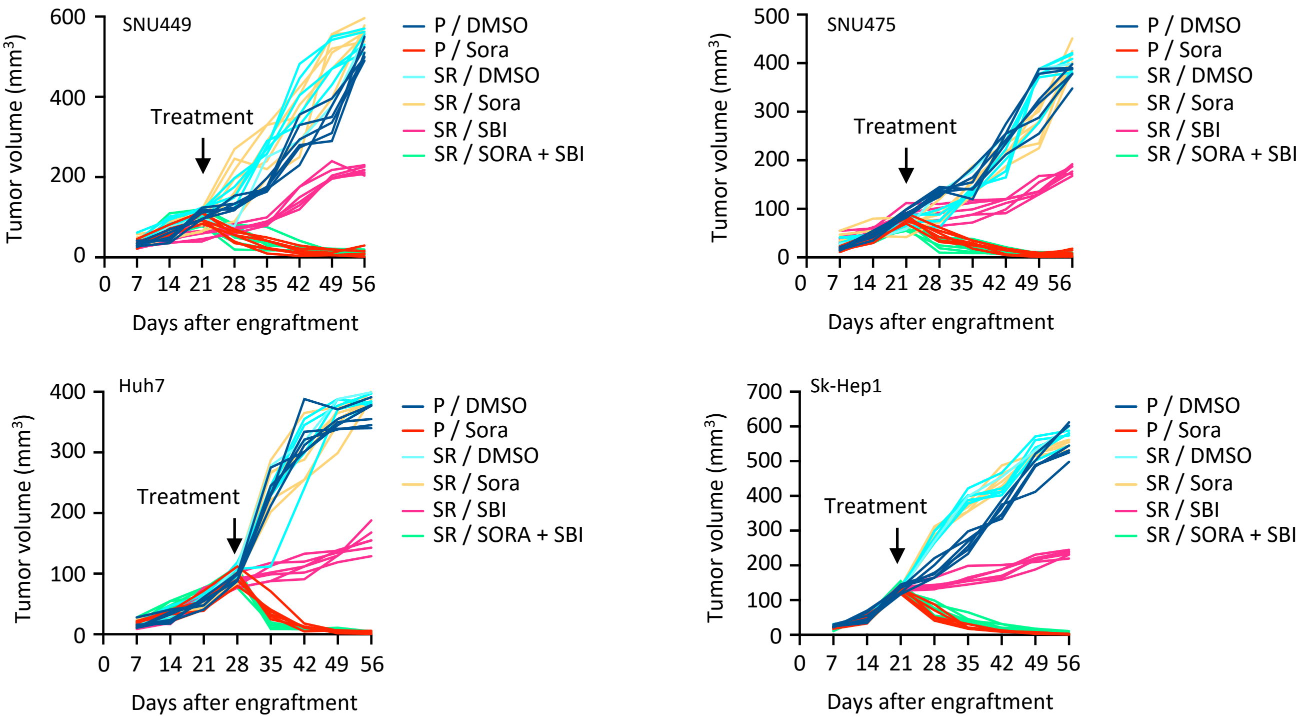

b

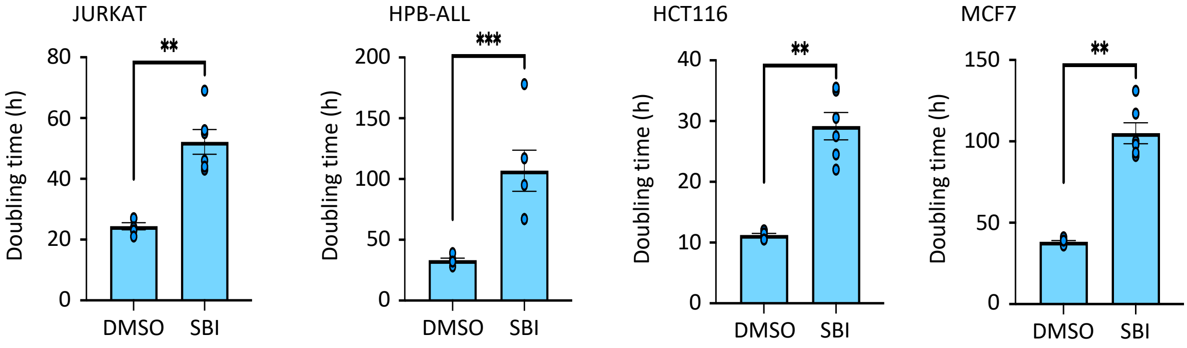

c

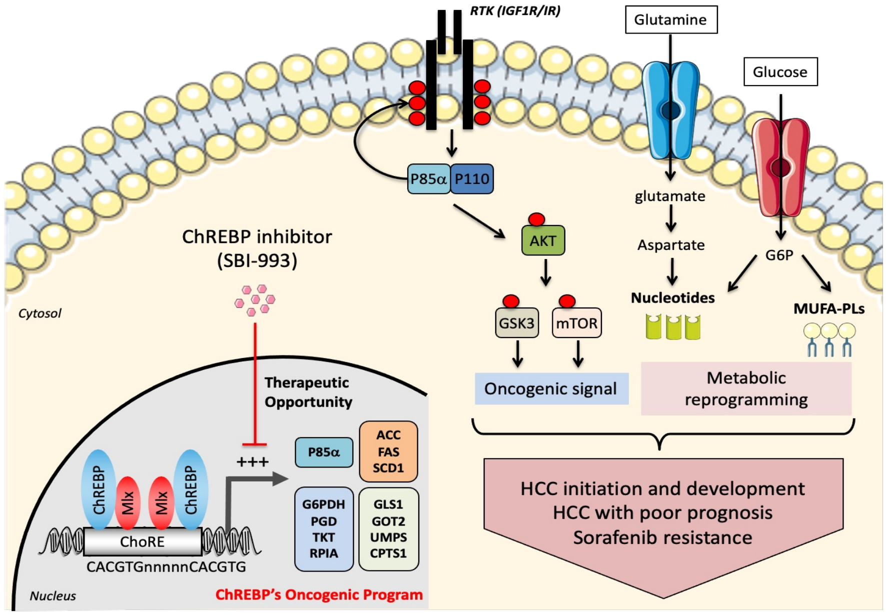

Figure. S17

Supplementary Figure 17. SBI-993 improves sorafenib efficacy during HCC treatment *in vivo*. **(a)** The xenograft mouse model was established by subcutaneous injection of 1×10^{7} parental or sorafenib resistant SNU449, SNU475, Huh7 or SK-Hep1 cells into male Nude *mice*. *Mice* were then daily treated with sorafenib alone (40 mg/kg) or in combination with SBI-993 (50 mg/kg) when tumor size reached at least 100 mm^3. The individual tumor growth kinetics of every mouse after different treatments are shown (n = 6 biologically independent *mice* per group). **(b)** Effect of SBI-993 treatment (40 µM) on JURKAT, HPB-ALL, HCT116 and MCF7 proliferation (n = 6 biologically independent *mice* per group). **(c)** Role of ChREBP during HCC initiation and development. ChREBP serves as a molecular link between growth signals (PI3K/AKT signaling) and acute control over determinant intertwined metabolic pathways that are determinant in neoplastic cell growth and proliferation. All error bars represent mean ± SEM. (b) Statistical analyses were made using unpaired two-sided Student's t-test. ** $P < 0.01$

Supplementary Table 1. Publicly available microarray Datasets analyzed for ChREBP expression and altered target genes in HCC patients

Number	Accession Number	Data compared *	Numbers of samples: Control	Numbers of samples: HCC	Main etiology reported	reference
1	GSE14520	Paired NT vs HCC	220	225	NAFLD/NASH, HBV	(Roessler et al., 2010)
2	GSE39791	Matched NT vs HCC	72	72	NAFLD/NASH, HBV	(Kim et al., 2014)
3	GSE57957	Adjacent NT vs HCC	39	39	NAFLD/NASH, HBV	(Mah et al., 2014)
4	GSE36376	Adjacent NT vs HCC	193	240	NAFLD/NASH, HBV	(Lim et al., 2013)
5	GSE60502	Adjacent NT vs HCC	18	18	NA	(Mas et al., 2009)
6	GSE14323	Normal liver vs HCC	19	38	HCV	(Wang et al., 2014)
7	GSE25097	Adjacent NT vs HCC	243	268	NAFLD/NASH, HCV, HBV	(Lamb et al., 2011)
8	GSE62232	Normal liver vs HCC	10	81	Mixed: Alcohol, NAFLD/NASH, HCV	(Schulze et al., 2015)
	LIHC dataset	Normal liver vs HCC	50	334	Mixed: Alcohol, NAFLD/NASH, HCV	
	LICA-FR dataset	Adjacent NT vs HCC	160	160	Mixed: Alcohol, NAFLD/NASH, HCV, HBV	
Total arrays			1024	1475		

HBV/HCV, hepatatis B/C virus; NAFLD, Non Alcoolic Fatty Liver Desease; NASH, Non Alocoolic Steatohepatatis; HCC, hepatocellular carcinoma.

NA, detail could not be accesed.

NT, non tumoral tissue.

* Description of the data compared as documented in the National Center for Biotechnology Information Gene Expression omnibus.

Differential expression was analyzed with GEO2R tool. The overall design for each data set can be found at https://www.ncbi.nlm.nih.gov/geo/.

Supplementary Table 2: Main clinical characteristics of the LIHC and LICA-FR datasets

For each clinical characteristic, % are given as proportion of samples with available data.

LICA-FR COHORT (n=160)

**Age**	Median 66	From 21 to 94 years old
**Gender**	M	126 (79%)
	F	34 (21%)
**Etiology**	Alcohol	63 (39%)
	Metabolic disease	37 (23%)
	HCV	30 (19%)
	HBV	30 (19%)
**Largest nodule diameter (mm)**	Median range	67 [12-210]
**Fibrosis (METAVIR score)**	F0-F1	75 (47%)
	F2-F3	32 (20%)
	F4	53 (33%)
**Edmonson grade**	I-II	48 (30%)
	III-IV	110 (70%)
	Not determined	2
**Vascular invasion**	yes	93 (58%)
	no	67 (42%)

LIHC COHORT (n=334)

**Age**	Median 61	From 16 to 90 years old
**Gender**	M	224 (67%)
	F	110 (33%)
**Etiology**	Alcohol	106 (32%)
	NAFLD	19 (6%)
	HCV	52 (16%)
	HBV	86 (26%)
**Fibrosis (Ishak score)**	0 - No Fibrosis	85 (36%)
	1,2 - Portal Fibrosis	35 (15%)
	3,4 - Fibrous Speta	28 (12%)
	5 - Nodular Formation and	9 (4%)
	6 - Established Cirrhosis	78 (33%)
	Not determined	99
**Histological grade**	G1	50 (15%)
	G2	160 (48%)
	G3	113 (34%)
	G4	7 (2%)
	Not determined	4
**Vascular invasion**	Macro	14 (5%)
	Micro	80 (29%)
	None	186 (66%)
	Not determined	54

Supplementary Table 3: Clinical characteristics of 12 HCC samples used for Western blots analysis

NUMBER	SEXE	AGE	BMI	NASH	STEATOSIS	Fibrosis stage	Metabolic syndrome	Hepatatis B	Hepatatis C	Alcohol intake	Edmonson Grade
1	M	68	24,9	0	0	0	no	yes	no	no	III-IV
2	F	83	22,7	1	1 < 5%	F4	no	no	yes	no	III-IV
3	M	83	26,3	1	1 < 10%	F3	yes	no	no	no	I-II
4	M	55	22,1	0	0	F4	no	yes	no	no	III-IV
5	M	62	22,1	1	0	F4	no	no	yes	no	III-IV
6	M	63	23,9	0	1 < 5%	F2	no	yes	no	no	I-II
7	M	70	28,3	0	1 > 40%	0	yes	no	yes	no	I-II
8	M	64	19,7	0	0	F3	no	no	yes	no	I-II
9	M	66	25,9	1	1 > 60%	F4	yes	no	no	no	III-IV
10	M	83	25,6	1	1 > 60%	F3	yes	no	yes	no	III-IV
11	M	76	24,3	0	1 > 40%	F4	yes	no	no	no	III-IV
12	M	55	18,3	0	1 < 5%	F1	no	no	no	yes	I-II
