# Supplementary material for: The transcription factor ChREBP Orchestrates liver carcinogenesis by coordinating the PI3K/AKT signaling and cancer metabolism
Source: Nat Commun. 2024 Feb 29;15:1879. doi: 10.1038/s41467-024-45548-w (PMC10904844; doi:10.1038/s41467-024-45548-w)
Supplement: Supplementary file 3 — Description of Additional Supplementary Files [file 41467_2024_45548_MOESM3_ESM.pdf]

Description of Additional Supplementary files

File name: Supplementary Data 1.
Description : Clinical and molecular characteristics of the LIHC dataset from oncomine. This set of data was download from the database on October 14, 2018

File name: Supplementary Data 2.
Description : Clinical and molecular characteristics of the LICA-FR dataset from oncomine. This set of data was download from the database on October 14, 2018

File name: Supplementary Data 3.
Description : ChREBP cancer gene signature depicting 324 genes that are upregulated in both our pre-tumoral and malignant model of ChREBP hyperactivation.

File name: Supplementary Data 4.
Description : GSEA analysis comparing ChREBP tumors with surrounding non-tumoral tissue.

File name: Supplementary Data 5.
Description : GSEA analysis comparing ChREBP tumors with surrounding non-tumoral tissue.

File name: Supplementary Data 6.
Description : gene expression in the GO: PI3K_signaling in the pre-tumoral and tumoral models.

File name: Supplementary Data 7.
Description : Pathway Analysis of potential ChREBP target genes identified by ChIP-seq and listed by KEGG.

File name: Supplementary Data 8.
Description : Metabolic genes consistently upregulated in all 10 HCC datasets - Total 285.

File name: Supplementary Data 9.
Description : Systematic toxicity evaluation during in vivo treatment of mice with SBI-993.

File name: Supplementary Data 10.
Description : Metabolomic and lipidomic analysis performed from adjacent non tumoral and ChREBP overexpressing tumors (n = 10 *mice* per group).
