# Supplementary material for: The transcription factor ChREBP Orchestrates liver carcinogenesis by coordinating the PI3K/AKT signaling and cancer metabolism
Source: Nat Commun. 2024 Feb 29;15:1879. doi: 10.1038/s41467-024-45548-w (PMC10904844; doi:10.1038/s41467-024-45548-w)
Supplement: Supplementary file 14 — Reporting Summary [file 41467_2024_45548_MOESM14_ESM.pdf]

nature research

Corresponding author(s): Renaud Dentin

Last updated by author(s): Dec 21, 2023

Reporting Summary

Nature Research wishes to improve the reproducibility of the work that we publish. This form provides structure for consistency and transparency in reporting. For further information on Nature Research policies, see our Editorial Policies and the Editorial Policy Checklist.

Statistics

For all statistical analyses, confirm that the following items are present in the figure legend, table legend, main text, or Methods section.

n/a	Confirmed	
☐	☒	The exact sample size (n) for each experimental group/condition, given as a discrete number and unit of measurement
☐	☒	A statement on whether measurements were taken from distinct samples or whether the same sample was measured repeatedly
☐	☒	The statistical test(s) used AND whether they are one- or two-sided<br>*Only common tests should be described solely by name; describe more complex techniques in the Methods section.*
☐	☒	A description of all covariates tested
☐	☒	A description of any assumptions or corrections, such as tests of normality and adjustment for multiple comparisons
☐	☒	A full description of the statistical parameters including central tendency (e.g. means) or other basic estimates (e.g. regression coefficient) AND variation (e.g. standard deviation) or associated estimates of uncertainty (e.g. confidence intervals)
☐	☒	For null hypothesis testing, the test statistic (e.g. F, t, r) with confidence intervals, effect sizes, degrees of freedom and P value noted<br>*Give P values as exact values whenever suitable.*
☒	☐	For Bayesian analysis, information on the choice of priors and Markov chain Monte Carlo settings
☒	☐	For hierarchical and complex designs, identification of the appropriate level for tests and full reporting of outcomes
☒	☐	Estimates of effect sizes (e.g. Cohen's d, Pearson's r), indicating how they were calculated

Our web collection on statistics for biologists contains articles on many of the points above.

Software and code

Policy information about availability of computer code

Data collection	A Chemidoc MP system instrument (Bio-Rad) was used for Western blots acquisition.<br>Lipidomic analysis was performed by gas-liquid chromatography on a FOCUS Thermo Electron system, using Zebron-1 (Phenomenex) fused silica capillary columns.<br>GC-MS analysis was performed with a Waters GCT Premier mass spectrometer fitted with an Agilent 6890 gas chromatograph and a Gerstel MPS2 autosampler. Data were collected using MassLynx 4.1 software.<br>Olympus BX51 microscope was used to acquire Ki67 and BrdU staining.<br>In vivo bioluminescent imaging was performed using a charge coupled device (CCD) camera (IVIS Spectrum system, PerkinElmer) and the resulting data were analyzed using the Living Image software (PerkinElmer).<br>Real-Time Cell Analysis (RTCA) was used for monitoring of cell proliferation using the iCELLigence device (Agilent Technologies, Inc).<br>Quantitative PCR was performed by Lightcycler 480 system (Roche).

Data analysis	Data analysis and statistical tests were done using Prism 8 (graphPad).<br>Quantification for clonogenic and BrDu assays were performed with ImageJ 1.80_172 software (http://rsb.info.nih.gov/ij/).<br>Results are expressed as mean ± SEM and were analyzed with analysis of variance.<br>Sample sizes (n) were reported in the corresponding figure legend.<br>All experiments were performed on at least three independent occasions.<br>No statistical method was used to predetermine sample size. After the normal distribution was confirmed with the Kolmogorov–Smirnov test, statistical comparisons between two groups were performed using a student's t test followed by Mann-Whitney post hoc test. Comparisons among multiple parameters were performed by two-way ANOVA followed by Bonferroni's post hoc comparisons. We did not estimate variations in the data. The variances are similar between the groups that are being statistically compared. In all cases, P values less than 0.05 were considered significant.

For manuscripts utilizing custom algorithms or software that are central to the research but not yet described in published literature, software must be made available to editors and reviewers. We strongly encourage code deposition in a community repository (e.g. GitHub). See the Nature Research guidelines for submitting code & software for further information.

Data

Policy information about availability of data

All manuscripts must include a data availability statement. This statement should provide the following information, where applicable:

- Accession codes, unique identifiers, or web links for publicly available datasets
- A list of figures that have associated raw data
- A description of any restrictions on data availability

All data used for expression analysis and GSEA are publicly available.
The datasets used for determining the expression levels of ChREBP within HCC tumors in human included (described in Supplementary Table 1):
1. GSE14520 (Roessler et al., 2010) https://www.ncbi.nlm.nih.gov/geo/query/acc.cgi?acc=GSE14520
2. GSE39791 (Kim et al., 2014) https://www.ncbi.nlm.nih.gov/geo/query/acc.cgi?acc=GSE39791
3. GSE57957 (Mah et al., 2014) https://www.ncbi.nlm.nih.gov/geo/query/acc.cgi?acc=GSE57957
4. GSE36376 (Lim et al., 2013) https://www.ncbi.nlm.nih.gov/geo/query/acc.cgi?acc=GSE36376
5. GSE60502 (Mas et al., 2009) https://www.ncbi.nlm.nih.gov/geo/query/acc.cgi?acc=GSE60502
6. GSE14323 (Wang et al., 2014) https://www.ncbi.nlm.nih.gov/geo/query/acc.cgi?acc=GSE14323
7. GSE6764 (Wurmbach et al., 2007) https://www.ncbi.nlm.nih.gov/geo/query/acc.cgi?acc=GSE6764
8. GSE62232 (Schulze et al., 2015) https://www.ncbi.nlm.nih.gov/geo/query/acc.cgi?acc=GSE62232
9. LIHC dataset (Oncomine database)
10. LICA-FR dataset (Oncomine Database)

Microarray analysis used for determining the role of ChREBP during liver carcinogenesis in mice can be found at the Gene Expression Omnibus database under accession number GSE159517 and GSE159518. Reported in the methods section of the manuscript (Data availability). There is no restriction on data availability, any additional information supporting the data within the paper and the findings of this study ara available from the corresponding author upon request.

Field-specific reporting

Please select the one below that is the best fit for your research. If you are not sure, read the appropriate sections before making your selection.

☒ Life sciences ☐ Behavioural & social sciences ☐ Ecological, evolutionary & environmental sciences

For a reference copy of the document with all sections, see nature.com/documents/nr-reporting-summary-flat.pdf

Life sciences study design

All studies must disclose on these points even when the disclosure is negative.

Sample size	No sample size calculations were performed. Sample size was determined to be adequate based on magnitude and consistency of measurable differences between groups. On the basis of previous studies using related experiments, the sample size has been determined to be sufficient to ensure reproductibility by performming at least three technical replicates of three biological independent experiments for in vitro studies. For in vivo experiments at least with 6 animals per group were included. Sample size are indicated in figure's legends for each panel.
Data exclusions	Data were only excluded for failed experiments in case of technical errors that occured during the excution of experiment. For experiments using hydrodynamic injections coupled with the used of the sleeping beauty transposon system, because two independent "hits" are required for tumor formation in FVB/N or C57BL/6 mice, only the hepatocytes that receive the two plasmids (transposon-based and transposase) will have the potential to form tumors. Therefore, mice that do not overexpressed the luciferase at day 7 were removed from the experimental cohort.
Replication	All experiments were repeated at least in 3 independent experiments to ensure reproductibility. Replication numbers (3 for each experiment) are reported in the figures, figure's legends or in the Methods section as required.
Randomization	No randomization method was used in the present study. Reported in the methods section of the manuscript. All mice analyzed were sex- and age-matched.
Blinding	For mice studies, tumor characterizations were scored in a "blinded" manner by experienced pathologists. For the rest of the study, the investigators were not blinded to allocation during experiments and outcome assessment. Reported in the methods section of the

manuscript.

Reporting for specific materials, systems and methods

We require information from authors about some types of materials, experimental systems and methods used in many studies. Here, indicate whether each material, system or method listed is relevant to your study. If you are not sure if a list item applies to your research, read the appropriate section before selecting a response.

Materials & experimental systems

n/a	Involved in the study
☐	☒ Antibodies
☐	☒ Eukaryotic cell lines
☒	☐ Palaeontology and archaeology
☐	☒ Animals and other organisms
☒	☐ Human research participants
☒	☐ Clinical data
☒	☐ Dual use research of concern

Methods

n/a	Involved in the study
☒	☐ ChIP-seq
☒	☐ Flow cytometry
☒	☐ MRI-based neuroimaging

Antibodies

Antibodies used

ChREBP (Novus, NB400-135), SCD1 (Cell Signaling, C12H5), LPK (Abcam, ab6191), ACC (Cell Signaling, 3662), FAS (Cell signaling, C20G5), G6PDH (Cell Signaling, 12263), GAPDH (Santa Cruz Biotechnology, FL-335), PKM2 (Cell Signaling, 4053), GOT1 (Cell Signaling, 344423), G6PDH (Cell Signaling, 8866), GOT2 (Novus Biologicals, NBP1-47469), HK2 (Cell Signaling, 2106), PGD (Novus Biologicals, NBP1-31589), RPIA (Novus Biologicals, NBP1-86214), ATP-Citrate Lyase (Cell Signaling, 4332), SCD1 (Cell Signaling, 2794), SLC1A5 (Novus Biologicals, NBP1-89327), Gls1 (Novus Biologicals, NBP1-89766), Gls2 (Novus Biologicals, NBP1-76544), GPT (Novus Biologicals, NBP1-89111), CAD (Cell Signaling, 11933), P-CAD Pser1859 (Cell Signaling, 12662), UMPS (Novus Biologicals, NBP1-85896), CTPS1 (Novus Biologicals, NBP1-52892), PRPS2 (Novus Biologicals, NBP1-31435), p(S473)-AKT (Cell signaling, D9E), Akt (Cell Signaling, 9272), P-p70S6K (Cell Signaling, 108D2), p70S6K (Cell Signaling, 49D7), p-GSK3b (Cell Signaling, D17D2), GSK3b (Cell Signaling, 27C10), PTEN (Cell Signaling, 9552), PI3 Kinase p85a (Cell Signaling, 13666), Afp (Cell Signaling, 4448), KI67 (Cell Signaling, 9449), Cyclin D1 (Cell Signaling, 2922). Cyclin B1 (Cell Signaling, 4138), Cyclin E (Cell Signaling, 4129), Cyclin A (Cell Signaling, 4656), PCNA (Cell Signaling, 2586), RNA PolII (Santa Cruz, sc899), HSP90 (Cell Signaling, 4874), H3K4me3 (Actif motif, 39915).

Validation

All the antibodies used in this study were commercials antibodies. Their validation can be found on the manufacturer's website using the provided catalog number.

Eukaryotic cell lines

Policy information about cell lines

Cell line source(s)

HepG2 (ATCC, # HB-8065), SNU-449 (ATCC, # CRL-2234), SNU-475 (ATCC, # CRL-2236), SK-HEP-1 (ATCC, # HTB-52), BNL CL.2 (ATCC, # TIB-73).

Authentication

HepG2, SNU-449, SNU-475, SK-HEP-1, BNL CL.2, were authenticated by ATCC through STR profiling. ChREBP Knockout cell lines or ChREBP overexpressing cell lines developped in the study were validated by Western blotting.

Mycoplasma contamination

All cell lines used in the study were tested on a regular basis for mycoplasma and were found to be mycoplasma free.

Commonly misidentified lines (See ICLAC register)

No commonly misidentified cell lines were used in this study?

Animals and other organisms

Policy information about studies involving animals; ARRIVE guidelines recommended for reporting animal research

Laboratory animals

6 weeks old male FVB/N mice, obtained from Charles River (France) were used to determine the pro-oncogenic function of ChREBP in the liver. ChREBP deficient male mice on a C57Bl6/J background, previously characterized in Iroz, A. et al (A Specific ChREBP and PPARalpha Cross-Talk Is Required for the Glucose-Mediated FGF21 Response. Cell Rep 21, 403-416, doi:10.1016/j.celrep.2017.09.065 (2017)), were used to determine the contribution of ChREBP to Myr-Akt mediated HCC initiation and development. For, our mouse xenograft models, male nude mice (Rj:NMRI-Foxn1nu/Foxn1nu) were purchase from Janvier lab (France). The endpoint of experiment was usually 6-8 weeks later after cell injection when the tumor volume was about 1.5 to 2 cm3. Mice were maintained in a 12-hr light/dark cycle with water and a standard diet (65% carbohydrate, 11% fat, and 24% protein).

Wild animals

No wild animals were used in this study.

Field-collected samples

No field-collected samples were used in this study.

Ethics oversight

All procedures using mice were carried out according to the French guidelines for the care and use of experimental animals (animal

authorization agreement number CEEA34.RD.082.12, Paris Descartes Ethical Committee).

Note that full information on the approval of the study protocol must also be provided in the manuscript.
